# A Systematic Review of Contemporary Randomized Trials in Cardiothoracic Surgery

**DOI:** 10.1016/j.atssr.2023.05.017

**Published:** 2023-06-12

**Authors:** N. Bryce Robinson, Mohamed Rahouma, Katia Audisio, Gianmarco Cancelli, Michelle Demetres, Giovanni Soletti, Irbaz Hameed, Leonard N. Girardi, Marc Ruel, Stephen E. Fremes, Mario Gaudino

**Affiliations:** 1 Department of Cardiothoracic Surgery, Weill Cornell Medicine, New York, New York; 2 Division of Cardiothoracic Surgery, Yale University School of Medicine, New Haven, Connecticut; 3 Division of Cardiac Surgery, University of Ottawa Heart Institute, Ottawa, Ontario, Canada; 4 Division of Cardiac Surgery, Schulich Heart Centre, Sunnybrook Health Sciences Centre, University of Toronto, Toronto, Ontario, Canada

## Abstract

**Background:**

This analysis was conducted to characterize contemporary randomized controlled trials (RCTs) in cardiothoracic surgery.

**Methods:**

We selected randomized controlled trials published in the journals with the highest impact factor in medicine, general surgery, and cardiothoracic surgery and published between 2008 and 2020. Trial characteristics as well as measures of reporting and quality were summarized and compared.

**Results:**

Ninety-three trials were included; 44 (47.3%) were prospectively registered and 14 (31.8%) had a discrepancy between the registered and published primary outcome. Most trials (n = 83 [89.1%]) used a superiority design, a composite primary outcome (n = 82 [88.2%]), and a major clinical event as the primary end point (n = 67 [72.0%]). Blinding was used infrequently, and most trials did not control for surgeon experience (n = 74 [79.5%]) or monitor the intervention (n = 90 [96.7%]). Twenty-four (25.8%) trials had high risk of bias. Twenty-one (27.3%) trials were funded by industry. A median 1.62% of patients (interquartile range, 0.00-3.70) crossed over between trial arms. Most trials reported a favorable outcome (n = 53 [58.9%]). For eligible trials, the median fragility index was 2.0 (interquartile range, 0.0-4.0), meaning the change of 2 patient outcomes would render the significant result insignificant. Spin, or distortion in reporting, was identified in 9 of 53 trials (17.0%). The median number of citations was 25 (10-56).

**Conclusions:**

Contemporary trials in cardiothoracic surgery are pragmatic with low rates of loss to follow-up and crossover. Few trials implemented measures to ensure quality of the intervention, and the presence of spin was infrequent.

**In Short**

▪ Contemporary randomized clinical trials in cardiothoracic surgery are small, with low rates of blinding and assessment for the uniform deliverability of the intervention.
▪ Rates of patient crossover and loss to follow-up were reassuringly low.
▪ As the number of randomized clinical trials in cardiothoracic surgery drops and access to funding becomes increasingly difficult, this analysis should serve to help guide future trial design to allow important clinical questions to be addressed using a high-quality methodology.

Randomized controlled trials (RCTs) in surgery face unique challenges, including nonstandardized delivery of the intervention, longer learning curve than in most other procedural fields, individual or collective equipoise, risk-averse surgical behaviors leading to indication bias, blinding, and scarce surgical research funding, among others.^1^ As a result, most evidence in surgery is derived from observational series.^2^ Although institutional and registry studies have an important role in the continued development of evidence, observational data carry a high risk of confounding and bias that may drive treatment effect rather than a true biologic effect.^3^

Even when RCTs in surgery are performed, they suffer from important limitations. High rates of crossover and inadequate delivery of the intervention, for example, have led many to call into question the results of some of the largest randomized trials published in cardiothoracic surgery.^4^^,^^5^ To date, there has been no systematic evaluation of the design and reporting of RCTs in cardiothoracic surgery. In this analysis, we evaluate contemporary RCTs in cardiothoracic surgery with a focus on trial design and reporting.

## Material and Methods

### Search Strategy

A literature search was performed by a medical librarian (M.D.) for randomized trials in cardiothoracic surgery published between January 1, 2008, and January 1, 2020, in the 2 journals with the highest impact factor in medicine (*The Lancet* and *The New England Journal of Medicine*), surgery (*JAMA Surgery* and *Annals of Surgery*), and cardiothoracic surgery (*The Journal of Thoracic and Cardiovascular Surgery* and *The Annals of Thoracic Surgery*). The full search strategy is available in Supplemental Table 1.

A surgical trial was one in which a surgical intervention, defined as any procedure performed by a trained surgical specialist with the goal of correction of deformities or defects, repair of injuries, or cure of certain diseases was tested in both the experimental and control arms. Trials evaluating nonsurgical interventions or endovascular and percutaneous interventions were excluded. In the case of multiple reports from 1 randomized trial, the report of the primary analysis was selected.

This study was prospectively registered on the international prospective register of systematic reviews (PROSPERO ID: CRD42020162797) and approved by the National Institute of Health Research.

### Extraction of Trial Data

Two authors screened all citations retrieved from the systematic search and extracted data on the basis of a previously defined methodology using a predefined data collection form. All discrepancies were resolved by a third author.

The following data were extracted from each eligible trial: journal and year of publication, 2018 journal impact factor as determined by Thomson Reuters–Clarivate Analytics, details of the surgical intervention including assessment of deliverability and control for learning curve effect, single-center or multicenter study, location, primary outcome, patients screened, percentage of screened patients enrolled, sample size, statistical power, treatment effect estimate, blinding and randomization use, details of the primary analysis, adjustment for multiple testing, trial sponsor details, conflicts of interest of the first and last authors, length of follow-up, number of patients lost to follow-up, crossover, and number of citations of the original study after publication as determined by Scopus/Web of Science.

Trials were also assessed for pragmatism using the Pragmatic-Explanatory Continuum Index Summary tool. In brief, a pragmatic trial is designed to assess an intervention in typical conditions, whereas an explanatory trial is one in which an intervention is tested in ideal conditions. The presence of spin and risk of bias were also assessed. Spin refers to the amount of distraction or misrepresentation of trial data despite a nonsignificant difference in the primary outcome. The Cochrane Risk of Bias tool is used to assess bias in randomized trials across 5 domains. The fragility index was calculated for each superiority-designed trial with a statistically significant dichotomous outcome. The fragility index is a calculation of a change in the smallest number of patient outcomes to render a formerly significant primary outcome nonsignificant. A detailed outline of data extraction methods is available in the Supplemental Methods.

The primary outcome for each trial was determined according to a published method and classified as a major or minor clinical end point based on a classification scheme designed according to previously published methodology (Supplemental Table 2).^6^

In studies with declared industry sponsorship, the conflict of interest for the first and last authors was extracted from the author disclosure statement. If co-first authors were listed, the conflict of interest for both was extracted.

### Statistical Analysis

Categorical variables were reported as counts and percentages. After assessment of normality by visual inspection and the Shapiro-Wilk normality test, continuous variables were reported as mean (SD) when normally distributed or as median (interquartile range [IQR]) when not. Based on normality of data, independent samples *t*-test or the Mann-Whitney *U* test was used to compare continuous variables. Categorical variables were compared using *χ*^2^ or Fisher exact tests. *P*-for-trend (linear regression) was used to evaluate variations during the course of the study period. Two-sided significance testing was used, and a *P* value < .05 was considered significant without adjustment for multiple testing. All analyses were performed using R (version 3.6.2; R Foundation for Statistical Computing) within RStudio.

## Results

### Trial Characteristics

The systematic search returned 4410 results, of which 93 met criteria for inclusion (Figure). Full details of the included trials and the reference list are provided in Supplemental Table 3.

**Figure:**
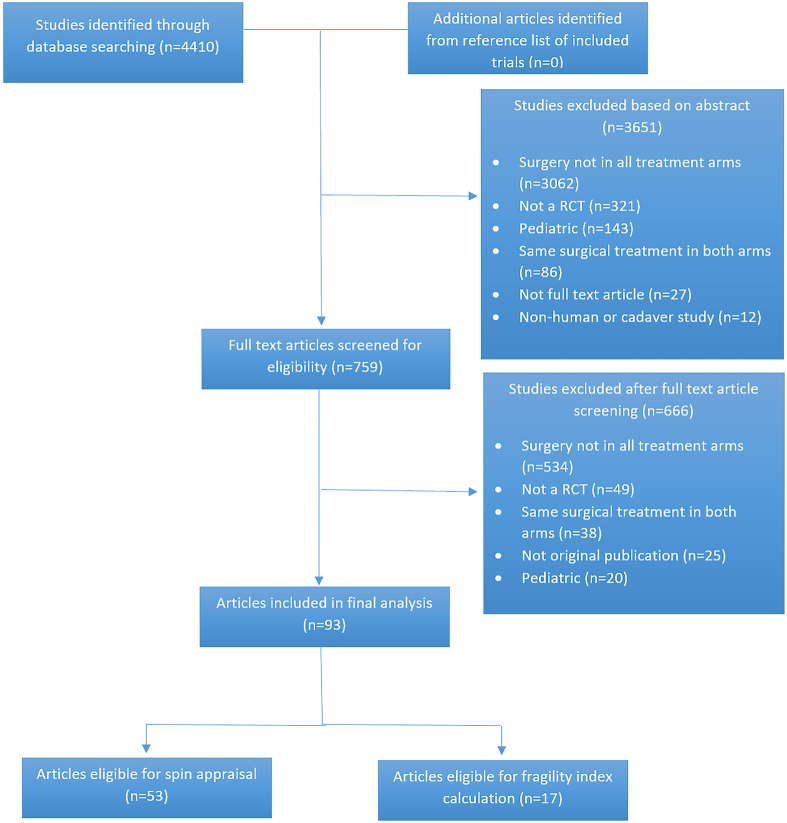
Preferred Reporting Items for Systematic Reviews and Meta-Analyses diagram. (RCT, randomized controlled trial.)

Most trials originated from Europe (n = 43 [46.2%]) and from North America 20 (21.5%). Thirty-nine (41.9%) trials were multicenter (Table 1). The number of included trials trended down during the course of the study period; however, this was not statistically significant (*P*-for-trend = .07; Supplemental Figure).

**Table 1:** Characteristics of Included Trials

Characteristic	No. (%)
Total No. of trials	93
Journal of publication	
*The Journal of Thoracic and Cardiovascular Surgery*	44 (47.3)
*The Annals of Thoracic Surgery*	32 (34.4)
*The New England Journal of Medicine*	10 (10.8)
*The Lancet*	3 (3.2)
*Annals of Surgery*	2 (2.2)
*JAMA Surgery*	2 (2.2)
Location	
Europe	43 (46.2)
North America	20 (21.5)
Asia	17 (18.3)
Australia	3 (3.2)
Africa	2 (2.2)
South America	1 (1.1)
Multiple continents	7 (7.5)
Multicentric trial	39 (41.9)

### Trial Design

Less than half of trials (n = 44 [47.3%]) were prospectively registered. Of the trials that were registered, 14 (31.8%) had a discrepancy between the registered and published primary outcome. Most trials (n = 83 [89.1%]) used a superiority design. Median power was 80% (IQR, 80-90), and trials were designed to detect a median estimated relative treatment effect of 34.9% (IQR, 20.9-50.0). Forty-six (49.4%) trials used intention-to-treat as the primary analysis. Most trials (n = 82 [88.2%]) used a composite primary outcome and a major clinical event as the primary end point (n = 67 [72.0%]). Time to event (n = 59 [63.4%]) was the most common type of primary outcome. Most used computer-generated randomization (n = 48 [51.6%]). Blinding was infrequent, with 19 (20.4%) trials blinding the outcome assessor only, 17 (18.3%) trials explicitly stating that no blinding was used, and 34 (36.5%) not giving any details related to blinding. Most trials did not control for surgeon experience (n = 74 [79.5%]) or monitor for implementation of the intervention (n = 90 [96.7%]) and provided limited details related to the intervention (n = 52 [55.9%]). Risk of bias was assessed in all trials; 17 (18.3%) had low risk of bias, 52 (55.9%) had some concerns, and 24 (25.8%) had high risk of bias. Seventy-seven trials (82.8%) reported external funding, of which 21 (27.3%) were funded by industry. In studies with industry funding, 11 first authors (52.3%) and 10 last authors (47.6%) reported a conflict of interest with the study sponsor. The mean Pragmatic-Explanatory Continuum Index Summary score was 3.49 (SD 0.55; Table 2).

**Table 2:** Trial Design

Variable	Frequency
Registration in trials registry	44 (47.3)
Discrepancy between registered and primary outcome	14 (31.8)
Superiority design	83 (89.1)
Noninferiority design	10 (10.9)
Power, %	80 (80-90)
Estimated relative treatment effect, %	34.9 (20.9-50.0)
Estimated treatment effect of trials with a major clinical end point as primary outcome, %	40.0 (20.0-50.0)
Estimated treatment effect of trials with a minor clinical end point as primary outcome, %	30.0 (25.0-50.0)
Intention-to-treat as the primary analysis	46 (49.4)
Use of composite primary outcome	82 (88.2)
Major clinical event as primary end point	67 (72.0)
Type of primary outcome	
Time to event	59 (63.4)
Quality of life	3 (3.2)
Other scales	31 (33.3)
Randomization	
Computer generated	48 (51.6)
Envelope	18 (19.4)
Random number table	7 (7.5)
Other	6 (6.5)
No details given	14 (15.1)
Blinding	
Outcome assessor only	19 (20.4)
None	17 (18.3)
Patient only	9 (9.7)
Patient and outcome assessor	8 (8.6)
Patient, outcome assessor, data analyst	5 (5.4)
Outcome assessor and data analyst	1 (1.1)
No details given	34 (36.5)
Control for surgeons’ experience	
None	74 (79.5)
Surgeons’ experience cutoff	14 (15.1)
Pretrial training	5 (5.4)
Monitoring of the intervention	
None	90 (96.7)
Site visit	2 (2.2)
Data monitoring of outcomes	1 (1.1)
Details of the experimental procedure	
None	8 (8.6)
Limited	52 (55.9)
Detailed	33 (35.5)
Risk of bias assessment	
Low risk	17 (18.3)
Some concerns	52 (55.9)
High risk	24 (25.8)
External funding	77 (82.8)
Industry funding	21 (27.3)
Industry sponsor involved in the analysis	16 (76.2)
Conflicts of interest	
Conflict of interest of first author with study sponsor	11 (52.3)
Conflict of interest of last author with study sponsor	10 (47.6)
Pragmatic-Explanatory Continuum Index Summary score, mean (SD)	3.49 (0.55)

Categorical variables are presented as number (percentage). Continuous variables are presented as median (interquartile range).

### Trial Implementation

Trials screened a median of 508 patients (IQR, 168-1224), enrolling a median of 129 (IQR, 57-246). Women included a median of 31% (IQR, 19-43) of the total study population, and the median number of women enrolled was 41 (IQR, 20-82). Race or ethnicity of study participants was reported in 10 (10.7%) trials. The median percentage of patients screened who were enrolled was 67.8% (IQR, 48.2-88.7). The median duration of follow-up was 20.0 months (IQR, 12.0-36.0). A median of 1.50 patients (IQR, 0.00-11.25) were lost to follow-up. The median fragility index was 2.0 (IQR: 0.0-4.0), and the fragility index minus lost to follow-up was 2 (IQR, 0-4). A median of 1.62% of patients (IQR, 0.00-3.70) crossed over between trial arms (Table 3).

**Table 3:** Trial Implementation

Variable	Median (IQR)
No. of patients screened	508 (168-1224)
Sample size	129 (57-246)
Percentage of women included	31 (19-43)
Race or ethnicity reported, n (%)	10 (10.7)
Duration of follow-up, months	20.0 (12.0-36.0)
Percentage of screened patients included	67.8 (48.2-88.7)
Patients lost to follow-up	1.50 (0.00-11.25)
Percentage sample size lost to follow-up	0.50 (0.00-7.45)
Fragility index	2.0 (0.0-4.0)
Fragility index minus lost to follow-up	2.0 (0.0-4.0)
No. of crossovers	2.00 (0.00-8.00)
Percentage of crossover	1.62 (0.00-3.70)

IQR, interquartile range, n, number of studies.

### Trial Reporting and Citations

Most trials reported a favorable outcome (n = 53 [58.9%]). Multiplicity was identified in 53 trials (56.9%), of which 7 (13.2%) made adjustment for multiple testing. Spin was identified in 9 of the 53 eligible trials for appraisal (17.0%). The median number of citations was 25 (10-56; Supplemental Table 4). *The New England Journal of Medicine* had the highest median citation rate (345 [IQR, 223-406]), and *The Annals of Thoracic Surgery* had the lowest (16 [IQR, 7-40]; Supplemental Table 5).

## Comment

In this analysis, we evaluated 93 contemporary randomized trials in cardiothoracic surgery published between 2008 and 2020. Trial registration was suboptimal, and for trials registered, a discrepancy between the registered and published primary outcome was identified in almost one-third. Blinding, control for the surgeon's experience, and formal assessment of the deliverability of the intervention were infrequently used. A quarter of trials had high risk of bias. Notably, trials were relatively pragmatic, and loss to follow-up and crossover rates were low. Rates of spin were also lower than those reported in analyses in other fields.^6^

Because of the limited number of RCTs in cardiothoracic surgery, it is critically important that trial design be improved to answer clinically important questions with high methodologic certainty. This is especially important as the number of funded cardiothoracic surgeon-scientists is declining. In fact, a 2022 analysis of National Institutes of Health funding found that among 10 surgical specialties included, only cardiothoracic surgery saw a decline in the number of funded surgeons, whereas all other specialties saw a net increase.^7^ As such, effort must be made to maximize the yield from the limited funding available.

Although progress has been made since cardiac surgery was identified by the National Heart, Lung, and Blood Institute as a gap area in 2004, there is still room for improvement. Two of the largest trials published to date in cardiothoracic surgery suffer from important methodologic limitations, and as such their results have been called into question by the surgical community. Initiated in 2004, the Arterial Revascularization Trial suffered a higher than expected crossover rate from the bilateral to single internal thoracic artery arm (14%) as well as a high rate of radial artery use in the single internal thoracic artery group (21.8%), which essentially moved those patients into the multiple arterial grafting group.^8^ Interestingly, we did not find a high rate of crossover in the trials included in this analysis. Surgeon training and monitoring of the intervention, however, were low and offer an opportunity for improvement in future trials to avoid the limitations of the Arterial Revascularization Trial.

Similar methodologic limitations exist in the Surgical Treatment for Ischemic Heart Failure (STICH) trial, in which the delivery of the intervention in the surgical ventricular reconstruction group was suboptimal, with a higher than expected median postoperative left ventricular end-systolic volume index and an overall volume reduction of just 13%.^9^

Our group has previously published on the characteristics of RCTs across multiple surgical fields including neurosurgery and general, orthopedic, transplant, and vascular surgery.^10^ Similar to this analysis, we found that contemporary RCTs in other surgical fields were small yet pragmatic, with excellent follow-up and low crossover between treatment arms. Few trials controlled for the intervention or assessed for quality, and many were unregistered, findings consistent with this analysis. To be sure, prospective trial registration is suboptimal across all medical subspecialties, with a recent analysis of 6788 RCTs finding that the pooled proportion of registered RCTs was 51% (95% CI, 44%-58%).^11^

There are areas in which the included trials are to be commended. Notably, the rate of spin (17%) was lower than that reported in previous analyses.^10^ Most trials used a major clinical end point as a primary outcome. Risk of bias was generally low and trial design was pragmatic. There are a number of areas for improvement, however, especially compared with trends noted in other surgical specialties. About half of trials in cardiothoracic surgery were registered, a rate that falls below that observed in general surgery trials (91.2%) included in our prior analysis. In addition, the fragility index in this analysis was low, with a change in status of just 2 patients from a nonevent to an event to render a significant difference nonsignificant. Whereas no cutoff value for fragility index exists, denoting a “fragile” trial from one that is not, trialists should work to design trials such that such a small change in patient outcomes will not affect the primary outcome.

This analysis should be interpreted in the context of several limitations. First, not all trials in cardiothoracic surgery published during the study period were captured by the search strategy in limited journals, and there is a potential that important studies were excluded. Second, we did not capture unpublished trials, and this analysis is therefore subject to publication bias. Third, by limiting the search strategy to the top 2 journals by impact factor in the selected fields, there is a possibility of selection bias. Finally, although they are established tools, the Pragmatic-Explanatory Continuum Index Summary score, fragility index, and spin have known limitations.^12^
