# Supplementary material for: A Systematic Review of Contemporary Randomized Trials in Cardiothoracic Surgery
Source: Ann Thorac Surg Short Rep. 2023 Jun 12;1(3):537–41. doi: 10.1016/j.atssr.2023.05.017 (PMC11708136; doi:10.1016/j.atssr.2023.05.017)
Supplement: Supplementary Tables [file mmc1.docx]

**Appendix**

**Supplementary Methods:**

*Assessment of Trial Pragmatism*

The Pragmatic Explanatory Continuum Index Summary (PRECIS-2) tool was developed to help determine if a randomized trial was more pragmatic or explanatory in its design.[1,2] Pragmatic trials are designed to answer the question “will this intervention work in typical conditions,” whereas explanatory trials are designed to test an intervention in “ideal conditions.” The PRECIS-2 tool is used to evaluate trial pragmatism in nine domains: eligibility criteria, recruitment, setting, organization, flexibility of intervention delivery, flexibility of adherence to the intervention, follow-up, primary outcome, and primary analysis. Two reviewers (NBR and IH) rated the level of pragmatism in each trial design domain using the following 5-point Likert scale: (1) very explanatory, (2) rather explanatory, (3) equally pragmatic/explanatory, (4) rather pragmatic, and (5) very pragmatic. Disagreements between reviewers were reconciled by a third reviewer (MG).

*Classification of trial results*

Trials were classified as “favorable” if, for at least one primary outcome among those defined in the protocol, the experimental therapy was significantly better than the control therapy (p <0.05 or a 95% confidence interval [CI] which excludes the null value) in superiority trials, the experimental therapy did not exceed the non-inferiority margin in non-inferiority trials, or the effects of the treatments differed by no more than the equivalence margin in equivalence trials.[4]

*Appraisal of Spin*

Studies reporting a non-significant difference in the primary outcome were eligible for the appraisal of spin. Briefly, spin is the presence and amount of distortion or misrepresentation of benefit despite a statistically non-significant difference for the primary outcome, or to distract the reader from statistically non-significant results.[5]

Following a previously described method,[5] the presence of spin was assessed in the following sections of the manuscript by two independent reviewers (NBR and IH) blinded to trial details: title, abstract results, main text results, discussion, abstract conclusions and main text conclusions. The spin strategies considered were: (1) a focus on secondary statistically significant results; (2) interpreting statistically non-significant results for the primary outcomes as showing treatment equivalence or comparable effectiveness; (3) claiming or emphasizing the beneficial effect of the experimental treatment despite statistically non-significant results; and (4) claiming or emphasizing non-inferiority despite a lack of pre-established non-inferiority boundaries or when data are inconclusive. Other spin strategies that are not classified according to this scheme were recorded and classified as “other”. The number of sections with spin was recorded and the extent of spin across a study was defined as the number of sections with spin in the entire article.

*Assessment of Multiplicity*

Each trial was assessed for the presence of multiple testing for the primary outcome, as well as any adjustment as previously reported.[6]

*Trial sponsorship*

Trials were classified as commercially-sponsored if they were either industry-initiated and/or sponsored, or were investigator-initiated studies that received commercial support. For commercially-sponsored trials, the body of the article, supplementary materials and original trial designs were additionally analyzed for the reporting of commercial or sponsor involvement in the trial design, conduct, analysis, or reporting.

*Assessment of discrepancies between the registered and published primary outcomes*

For prospectively registered trials clearly describing the primary outcome in the registry, we assessed for a discrepancy between the registered and reported outcome. Consistent with previous definitions,[7,8] major discrepancies were defined as: (1) a pre-specified primary outcome in the trial registration protocol reported as a secondary outcome in the final published article; (2) a published primary outcome described as a secondary outcome in the registry; (3) a pre-specified primary outcomes in the trial registration not reported in the published article; (4) a new primary outcome introduced in the published article; and (5) different timing of assessment of the primary outcome in the registered protocol and published article.[8]

*Calculation of the Fragility Index*

Superiority-design trials reporting at least one statistically significant dichotomous primary outcome (p <0.05 or a 95% CI excluding the null value) were eligible for calculation of the Fragility Index (FI).^6^  Results for each outcome were entered in a 2x2 contingency table following which the p-value for each outcome was calculated using the two-sided Fisher’s exact test. Individual participants were then iteratively shifted one at a time in the lower-incidence treatment group from “non-event” to “event” and the p-value for the 2x2 table was re-calculated. The FI for a given outcome was reported as the smallest number of patients required to render the re-calculated p-value non-significant (≥0.05). Lower values indicate less robust results. The difference between the FI and the number of patients lost to follow-up was calculated according to the methods used by Mazzinari et al.[9]

*Risk of Bias Assessment*

All trials were eligible for assessment of risk of bias using the Cochrane Risk of Bias Tool Version 2 (RoB 2).[10] The RoB 2 tool assesses for bias introduced to the results of randomized trials across five domains: bias arising from the randomization process; bias due to deviations from intended interventions; bias due to missing outcome data; bias in measurement of the outcome; and bias in selection of the reported result. For each domain, risk of bias was categorized into one of three categories: low, some concerns, or high. A study was classified as low risk if all domains were categorized as low risk, some concerns if at least one domain was categorized as some concerns but not found to be high risk for any domain, and high risk if at least one domain was categorized as high risk or if the study was judged to have some concern across multiple domains in a way that substantially impacted trial results.

**Supplementary Table 1**: Search strategy

| **Medicine** |
| --- |
| ***The New England Journal of Medicine***  Line # \| Search \| # of results  1 "randomized controlled trial".pt. 495639  2 (random$ or placebo$ or single blind$ or double blind$ or triple blind$).ti,ab.  1186604  3 (retraction of publication or retracted publication).pt. 14645  4 or/1-3 1298384  5 (animals not humans).sh. 4615830  6 ((comment or editorial or meta-analysis or practice-guideline or review or letter) not "randomized controlled trial").pt. 4409095  7 (random sampl$ or random digit$ or random effect$ or random survey or random regression).ti,ab. not "randomized controlled trial".pt. 84751  8 4 not (5 or 6 or 7) 951334  9 "new england journal of medicine".jn. 78687  10 limit 9 to yr="2008 - 2018" 16267  11 8 and 10 1477  UPDATE:  12 limit 9 to yr="2019" 1497  13 8 and 12 173  TOTAL: 1650 |
| ***The Lancet***  1 "randomized controlled trial".pt. 495639  2 (random$ or placebo$ or single blind$ or double blind$ or triple blind$).ti,ab.  1186604  3 (retraction of publication or retracted publication).pt. 14645  4 or/1-3 1298384  5 (animals not humans).sh. 4615830  6 ((comment or editorial or meta-analysis or practice-guideline or review or letter) not "randomized controlled trial").pt. 4409095  7 (random sampl$ or random digit$ or random effect$ or random survey or random regression).ti,ab. not "randomized controlled trial".pt. 84751  8 4 not (5 or 6 or 7) 951334  9 lancet.jn. 137172  10 limit 9 to yr="2008 - 2018" 17342  11 8 and 10 1222  UPDATE:  12 limit 9 to yr="2019" 1693  13 8 and 12 128  TOTAL: 1350 |
| **Surgery** |
| ***Annals of Surgery***  1 "randomized controlled trial".pt. 495639  2 (random$ or placebo$ or single blind$ or double blind$ or triple blind$).ti,ab.  1186604  3 (retraction of publication or retracted publication).pt. 14645  4 or/1-3 1298384  5 (animals not humans).sh. 4615830  6 ((comment or editorial or meta-analysis or practice-guideline or review or letter) not "randomized controlled trial").pt. 4409095  7 (random sampl$ or random digit$ or random effect$ or random survey or random regression).ti,ab. not "randomized controlled trial".pt. 84751  8 4 not (5 or 6 or 7) 951334  9 "annals of surgery".jn. 30910  10 limit 9 to yr="2008 - 2018" 4883  11 8 and 10 515  UPDATE:  12 limit 9 to yr="2019" 758  13 8 and 12 79  TOTAL: 594 |
| ***JAMA Surgery***  1 "randomized controlled trial".pt. 495639  2 (random$ or placebo$ or single blind$ or double blind$ or triple blind$).ti,ab.  1186604  3 (retraction of publication or retracted publication).pt. 14645  4 or/1-3 1298384  5 (animals not humans).sh. 4615830  6 ((comment or editorial or meta-analysis or practice-guideline or review or letter) not "randomized controlled trial").pt. 4409095  7 (random sampl$ or random digit$ or random effect$ or random survey or random regression).ti,ab. not "randomized controlled trial".pt. 84751  8 4 not (5 or 6 or 7) 951334  9 "jama surgery".jn. 2469  10 limit 9 to yr="2008 - 2018" 1973  11 8 and 10 68  UPDATE:  12 limit 9 to yr="2019" 486  13 8 and 12 25  TOTAL: 93 |
| **Thoracic surgery** |
| ***The Journal of Thoracic and Cardiovascular Surgery***  1 "randomized controlled trial".pt. 495639  2 (random$ or placebo$ or single blind$ or double blind$ or triple blind$).ti,ab.  1186604  3 (retraction of publication or retracted publication).pt. 14645  4 or/1-3 1298384  5 (animals not humans).sh. 4615830  6 ((comment or editorial or meta-analysis or practice-guideline or review or letter) not "randomized controlled trial").pt. 4409095  7 (random sampl$ or random digit$ or random effect$ or random survey or random regression).ti,ab. not "randomized controlled trial".pt. 84751  8 4 not (5 or 6 or 7) 951334  9 "journal of thoracic & cardiovascular surgery".jn. 28100  10 limit 9 to yr="2008 - 2018" 9569  11 8 and 10 334  UPDATE:  12 limit 9 to yr="2019" 1639  13 8 and 12 34  TOTAL: 368 |
| ***Annals of Thoracic Surgery***  1 "randomized controlled trial".pt. 495639  2 (random$ or placebo$ or single blind$ or double blind$ or triple blind$).ti,ab.  1186604  3 (retraction of publication or retracted publication).pt. 14645  4 or/1-3 1298384  5 (animals not humans).sh. 4615830  6 ((comment or editorial or meta-analysis or practice-guideline or review or letter) not "randomized controlled trial").pt. 4409095  7 (random sampl$ or random digit$ or random effect$ or random survey or random regression).ti,ab. not "randomized controlled trial".pt. 84751  8 4 not (5 or 6 or 7) 951334  9 "annals of thoracic surgery".jn. 35979  10 limit 9 to yr="2008 - 2018" 12309  11 8 and 10 329  UPDATE:  12 limit 9 to yr="2019" 991  13 8 and 12 26  TOTAL: 355 |

**Supplementary Table 2**: Major and minor clinical endpoints

|  | Primary endpoint |
| --- | --- |
| Major | - Need for transfusion - Chylothorax - Deep sternal wound infection - Disease free survival - Myocardial infarction - Neurocognitive decline - Survival - Pneumonia - Renal replacement therapy - Rehospitalization - Reoperation or device removal - Repeat revascularization - Stroke |
| Minor | - Atrial fibrillation - Anastomotic graft patency as assessed by angiography - Cosmetic outcomes - Cross clamp and cardiopulmonary bypass times - Duration of air leak - Efficacy of blood oxygenation - Freedom from structural valvular degeneration - Gas emboli on transesophageal echocardiography - Graft hyperplasia (as measured by ultrasound) - Harvest dysesthesia - Inflammatory markers - Length of stay - Left ventricular dimension changes - Left ventricular remodeling as assessed by echocardiography - Left ventricular end-systolic volume index - Myocardial enzyme release - Number of HITS on transcranial Doppler ultrasonography - Occurrence of moderate to severe functional tricuspid regurgitation - Pain and analgesia requirement - Postoperative air leak - Quality of life scores - Recurrence of pneumothorax - Return to spontaneous rhythm - Success of left atrial appendage closure assessed by echocardiography - Surgical time - Spirometry measurements - Staple height - Sternal blood flow - Sternal malunion - Sternal healing - Valvular hemodynamics and orifice size |

Footnote: In trials using a composite outcome, if any major clinical event was included in the composite the outcome was classified as major.

**Supplementary Table 3**: Summary of the included trials

| Trial number | Name of trial | Year | Journal | Aim | Primary outcome | Primary results | Number of patients screened | Number of patients included |
| --- | --- | --- | --- | --- | --- | --- | --- | --- |
| 1 | Chen et al[11] | 2012 | Annals of Surgery | To compare the efficacy and safety between apical pleurectomy and pleural abrasion with minocycline in primary spontaneous pneumothorax (PSP) with high recurrence risk | Rate of ipsilateral recurrence after the operation | After a mean follow-up of 26.1 months, recurrent ipsilateral pneumothorax occurred in 3 patients (3.8%) in the pleurectomy group and 3 patients (3.8%) in the abrasion/minocycline group | 369 | 160 |
| 2 | FOREseal[12] | 2017 | Annals of Surgery | To determine the efficacy of  alginate staple-line reinforcement of fissure openings as compared with stapling alone, with or without tissue sealant or glue, in reducing the incidence and duration of air leakage after pulmonary lobectomy for malignancy | Duration of postoperative air leak (in days) | The primary endpoint of air leak duration was not different between the 2 groups: 1 day (range: 0–2 d) in the FOREseal group and 1 day (range: 0–3 d) in the control group (P=0.8357) | 611 | 380 |
| 3 | Cerfolio et al[13] | 2008 | Annals of Thoracic Surgery | To evaluate if leaving the intercostal muscle flap intact reduces postoperative pain | Numeric pain score at postoperative weeks 3, 4, 8, and 12, survival, Readmit ≤30 days post-op, Prescription pain med use, Receiving adjuvant treatment, Return to baseline activities, Satisfied with procedure/care | Intrahospital pain scores were similar; however, at postoperative weeks 3, 4, 8, and 12, the D group had significantly lower mean numeric pain scores and was using fewer analgesics (p < 0.05 for all). At 12 weeks, patients in the D group were more likely to have returned to baseline activity (p=0.002). | 263 | 160 |
| 4 | Li et al[14] | 2008 | Annals of Thoracic Surgery | To measure the impact of limited denervation on compensatory sweating while performing endoscopic thoracic sympthectomy | Symptom resolution, postoperative complication, levels of satisfaction, and severity of compensatory sweating | The postoperative complications were minor, and Horner’s syndrome was not detected in either group. The frequency of mild and moderate compensatory sweating was not significantly different between the two groups, but the incidence of severe compensatory sweating was significantly lower after T3 sympathectomy (3% versus 10%). As for satisfaction rate, group T3 was superior to group T2–4 (96.6% versus 89.6%). The rate of symptom resolution was 100%, and no recurrence was found in either group | NR | 232 |
| 5 | Schimmer et al[15] | 2008 | Annals of Thoracic Surgery | To compare two surgical techniques with respect to the occurrence of SWI in patients with an increased risk | The rate of sternal dehiscence as well as the occurrence of superficial sternal wound infections and deep sternal wound infections | The rate of sternal dehiscence, superficial sternal wound infections, and deep sternal wound infections (conventional technique 2.5%, 3.4%, 2.5%; and Robicsek 3.7%, 5.6%, 3.7%) did not differ between the groups | NR | 815 |
| 6 | Krishnamoorthy et al[16] | 2009 | Annals of Thoracic Surgery | To compare results of skin closure using Dermabond and subcuticular sutures after coronary artery bypass grafting (CABG) | Cosmetic outcomes at weeks 1 and 6 | Patients in the Dermabond group also reported superior cosmetic outcome at weeks 1 (p < 0.001) and 6 (p=0.001) and improved patient satisfaction (p < 0.001) | NR | 106 |
| 7 | Suri et al[17] | 2009 | Annals of Thoracic Surgery | To investigate the hemodynamic performance of two widely used, stented xenograft biologic prostheses and to determine the effects of valve hemodynamics on regression of LV hypertrophy 1 year after surgery | Transprosthetic gradient, aortic valve orifice area, and LV mass | The mean aortic valve gradient at dismissal was 19.4 mm Hg (MM) versus 13.5 mm Hg (EP; p < 0.0001), and at 1 year was 20.4 mm Hg versus 13.4 mm Hg (p < 0.0001). During the first year after implantation, both groups demonstrated similar regression of LV mass index (MM, 32.4 g/m^2^ versus EP, 27.0 g/m^2^; p=0.40) | NR | 152 |
| 8 | Allama[18] | 2010 | Annals of Thoracic Surgery | To evaluate the effect of nondivided intercostal muscle flap and intracostal sutures (protecting the intercostal nerves from trauma) on early postoperative pain were investigated, comparing this technique with traditional pericostal sutures | Pain, which was measured by a blinded trained physician using the numeric rating scale from 0 to 10 (0 = no pain, 10 = extreme pain) postoperatively daily until the seventh day, at 1, 3, and 6 months | Postoperative pain score throughout the first week was significantly lower in the patients in the intercostal muscle flap group, who had also a significantly earlier postoperative ambulation and return to normal daily activities, and received significantly lower doses of postoperative analgesics. After 1 month, patients in the intercostal muscle flap group had a significantly lower pain score and use of analgesics. After 3 months, pain score was not significantly different between both groups, but the use of analgesics was significantly lower in the intercostal muscle flap group. After 6 months, no significant difference was present between both groups with regard to pain score or the use of analgesics | 168 | 120 |
| 9 | Yousefnia et al[19] | 2010 | Annals of Thoracic Surgery | To investigate the feasibility of performing papillary muscle repositioning (PMR) for subvalvular-sparing mitral valve replacement  procedures in patients with ischemic mitral regurgitation  and to determine the early and late effects of this procedure on the clinical outcome and left ventricular  mechanics | No primary outcome explicitly stated | Not reported | NR | 50 |
| 10 | Aykut et al[20] | 2011 | Annals of Thoracic Surgery | To compare the incidence of sternal dehiscence after prophylactic sternal weave and figure-of-eight suturing in diabetic obese patients undergoing coronary artery bypass grafting (CABG) | Sternal dehiscence according to clinical examination and chest radiography | There were 8 cases of sternal dehiscence documented: 7 in group A (figure of eight group) and 1 in group B (sternal weave group). Sternal dehiscence was significantly lower in group B (p < 0.05) | NR | 150 |
| 11 | Baumgartner et al[21] | 2011 | Annals of Thoracic Surgery | To compare sympathicotomy over the second (R2) vs third (R3) costal head relative to these variables in patients with massive palmar hyperhidrosis | Level of recurrence, dramatic failures, Compensatory Hyperhidrosis severity scale, subjective change in plantar sweating at 6 months and 1 year | Sympathicotomy at R2 failed to cure palmar hyperhidrosis in 5 of 122 (4.1%) extremities, but only 2 (1.6%) were to a truly profound dripping level of recurrence. Sympathicotomy at R3 failed to cure palmar hyperhidrosis in 5 of 120 extremities (4.2%), and all were dramatic failures with dripping recurrent sweating | NR | 121 |
| 12 | Lai et al[22] | 2011 | Annals of Thoracic Surgery | To evaluate the preventive effect of thoracic duct mass ligation on postoperative chylothorax | Occurrence rates of chylothorax | Chylothorax occurred in 8 patients, giving an incidence of 1.2%. In the preservation group, chylothorax occurred in 7 patients (2.1%), and in the prevention group, 1 case of chylothorax was found (0.3%). The incidence of postoperative chylothorax was significantly lower in the prevention group | 746 | 653 |
| 13 | Mannacio et al[23] | 2011 | Annals of Thoracic Surgery | To evaluate the flow outcome of the skeletonized versus pedicled left internal mammary artery | LIMA flow before papaverine application | Skeletonized left internal mammary arteries demonstrated better flow capacity at rest and during adenosine recruitment perioperatively and at all time points of follow-up | NR | 200 |
| 14 | Aye et al[24] | 2012 | Annals of Thoracic Surgery | To compare the effectiveness of Laparoscopic Hill repair LHR against the gold standard laparoscopic  Nissen fundoplication | Clinical recurrence as evidenced by reoperation for failure or resumption of antisecretory medication | Two LNF and two LHR required reoperation for failed repair | NR | 111 |
| 15 | Licht et al[25] | 2012 | Annals of Thoracic Surgery | To compare rib-oriented (R2 vs R2–R3) sympathicotomy for isolated facial blushing | QOL, local effect on facial blushing, and side effects | QOL increased significantly in all social and mental domains in both groups. Overall, 85% of the patients had an excellent or satisfactory result, with no significant difference between the R2 procedure and the R2–R3 procedure. Mild recurrence of facial blushing occurred in 30% of patients within the first year. One patient experienced Horner’s syndrome. Compensatory sweating occurred in 93% of patients, gustatory sweating 36%, and dry hands in 66%; 13% of patients regretted the operation despite thorough preoperative selection and information | NR | 100 |
| 16 | Raman et al[26] | 2012 | Annals of Thoracic Surgery | To evaluate if sternal reconstruction after median sternotomy using rigid fixation with plates may improve bone healing and reduce pain when compared with wire cerclage | Sternal union, pain, and function | Sternal healing was superior in rigid plate fixation patients at both 3 and 6 months. Pain scores and narcotic usage were lower in rigid plate fixation patients. Significant differences in pain scores were observed at 3 weeks for total pain (p=0.020) and pain with coughing (p=0.0084) or sneezing (p=0.030) | NR | 140 |
| 17 | Birla et al[27] | 2013 | Annals of Thoracic Surgery | To compare the clinical performance of 2 stented porcine aortic bioprostheses: the Carpentier-Edwards supraannular aortic valve (CE-SAV) from Edwards Lifesciences and the Mosaic valve from Medtronic Corp | Freedom from SVD echocardiography at 1, 5, and 10 years after implantation | There were no statistically significant differences between the 2 groups in terms of structural valve deterioration (SVD) (p=0.16), paraprosthetic leak (p=0.13), thromboembolism (p=0.25), endocarditis (p=0.68), and freedom from reoperation at 5 years (p=0.27) | NR | 403 |
| 18 | Dreifaldt et al[28] | 2013 | Annals of Thoracic Surgery | To compare the patency of no-touch saphenous vein with that  of radial artery grafts | Patency of grafts at follow-up | The patency of grafts for no-touch saphenous vein and radial artery was 94% versus 82% (p=0.01), respectively. The patency of coronary arteries grafted with no-touch saphenous vein and radial artery grafts was 95% versus 84% (p=0.005), respectively | 1678 | 108 |
| 19 | Bolotin et al[29] | 2014 | Annals of Thoracic Surgery | To evaluate the safety and efficacy of a novel aortic cannula producing simultaneous forward flow and  backward suction for extracting solid and gaseous emboli from the ascending aorta and aortic arch upon their intraoperative release | The volume of new brain lesions measured by diffusion-weighted magnetic resonance imaging (DW-MRI), performed preoperatively and postoperatively | The volume of new brain lesion for the treatment group was (mean ± standard error of the mean) 44.00 ± 64.00 versus 126.56 ± 28.74 mm^3^ in the control group (p=0.004). Of the treatment group, 41% demonstrated new postoperative lesions versus 66% in the control group (p=0.03) | NR | 66 |
| 20 | Lee et al[30] | 2014 | Annals of Thoracic Surgery | To clarify whether an additional coverage procedure on the staple line after thoracoscopic bullectomy prevents postoperative recurrence compared with additional pleurodesis | Rates of ipsilateral 1-year recurrence rate and recurrence requiring intervention (RRI), defined as a postoperative recurrent pneumothorax large enough to need closed thoracostomy or reoperation | After a median follow-up of 19.5 months, the postoperative 1-year recurrence rate was 9.5% in the coverage group and 10.7% in the pleurodesis group. The 1-year recurrence rate requiring intervention was 5.8% in the coverage group and 7.8% in the pleurodesis group | NR | 1414 |
| 21 | Min et al[31] | 2014 | Annals of Thoracic Surgery | To investigate the effectiveness of mechanical pleurodesis after thoracoscopic treatment of primary spontaneous pneumothorax | The recurrence rate of the thoracoscopic wedge resection and mechanical pleurodesis (WR+MP) group | Postoperative recurrence rate did not significantly differ between groups (log-rank test p=0.791; Breslow test p=0.722) | NR | 305 |
| 22 | CADENCE-MIS[32] | 2015 | Annals of Thoracic Surgery | To compare the outcomes for  MIS-RDAVR with those of conventional FS-AVR | Cross-clamp time and CPB time | MIS-RDAVR was associated with significantly reduced aortic crossclamp times compared with FS-AVR (41.3 ± 20.3 vs 54.0 ± 20.3 minutes, p < 0.001), although cardiopulmonary bypass times were similar (68.8 ± 29.0 vs 74.4 ± 28.4 minutes, p=0.21) | NR | 100 |
| 23 | VEST[33] | 2015 | Annals of Thoracic Surgery | To investigate whether external stenting inhibits SVG diffuse intimal hyperplasia 1 year after coronary artery bypass graft surgery | SVG intimal hyperplasia (mean area) assessed by intravascular ultrasonography at 1 year | Overall SVG failure rates did not differ significantly between the two groups (30% stented versus 28.2% nonstented SVG, p=0.55). The SVG mean intimal hyperplasia area, assessed in 43 SVGs, was significantly reduced in the stented group (4.37 ± 1.40 mm^2^) versus nonstented group (5.12 ± 1.35 mm^2^, p=0.04) | NR | 30 |
| 24 | Baumbach et al[34] | 2016 | Annals of Thoracic Surgery | To investigate minimally invasive extracorporeal circulation (MECC) in coronary operation | Procedural and postoperative outcomes, including the levels of inflammatory factors (procalcitonin, interleukin (IL)-6, IL-8, and IL-10), tumor necrosis factor-α (TNF-α), and interferon-gamma (IFN-γ)) | Hospital mortality (n=1 versus n= 3; p=0.339) and other complications were similar. Hemoglobin level (111.9 ± 19.0 g/L versus 103.8 ± 14.6 g/L; p=0.001), the number of packed red blood cells (PRBCs) (1.1 ± 1.9 versus 1.7 ± 1.8; p=0.003), the levels of ILs (IL-6, 194.0 ± 131.8 pg/mL versus 289.2 ± 62.5 pg/mL; p=0.020; IL-8, 38.1 ± 27.3 pg/ mL versus 45.8 ± 43.4 pg/mL; p=0.012; IL-10, 29.0 ± 123.9 pg/mL versus 49.9 ± 85.6 pg/mL; p=0.012), TNF-a (3.8 ± 6.7 ng/mL versus 10.8 ± 47.7 ng/mL; p=0.049), and IFN-g (1.9 ± 1.9 pg/mL versus 4.5 ± 2.7 pg/mL; p=0.027) were in favor of patients in the MECC group | NR | 200 |
| 25 | Nguyen et al[35] | 2016 | Annals of Thoracic Surgery | To compare induction of reactive oxygen species (ROS) and activation of nuclear factor (NF)-kB, p38 mitogen-activated protein kinase (MAPK) within leukocytes, and leukocyte accumulation in cantharidin-induced blisters in patients exposed to miniaturized CPB (mCPB) and those who underwent conventional CPB  (cCPB) | ROS induction levels in granulocytes | ROS in lymphocytes were elevated in cCPB compared with mCPB (p < 0.01), whereas ROS in granulocytes and monocytes were similar between groups | 50 | 26 |
| 26 | Fiorelli et al[36] | 2017 | Annals of Thoracic Surgery | To evaluate whether cardiac autonomic changes could be associated with different extent of  sympathetic nerve resection in the management of essential palmar hyperhidrosis | To evaluate whether the changes in HRV could be associated with the extent of sympathetic resection. In each group, HRV was evaluated 7 day before ETS and 24 hours and 1, 3, and 6 months later, using a 24-hour Holter recording | In both groups, we observed a significant increase (p < 0.05) of vagal activity measurements as root mean square of the successive differences of heart period; proportion of adjacent  normal R-R intervals >50 ms; high frequency; and a significant decrease (p < 0.05) of adrenergic activity variables as heart rate, low frequency, and the ratio between low frequency and high frequency during daytime, nighttime, and 24-hour periods. These changes were significantly more evident (p < 0.05) in the sympathectomy group than in the sympathicotomy group | NR | 60 |
| 27 | Okami et al[37] | 2017 | Annals of Thoracic Surgery | To evaluate flat face with equal height staples and the stepped face with graduated height staples stapling ability in lobectomy | Scores of the staple formation at the lobar bronchial stump | The case scores were significantly lower in the equal height staples than in the graduated height staples (2.17 versus 2.88, p=0.0003) respectively | 69 | 61 |
| 28 | Pettersen et al[38] | 2017 | Annals of Thoracic Surgery | To evaluate the impact of pedicled veins on duration of operations, leg wound infections, and postoperative bleeding | Vein graft patency 6 months and 5 years | No significant difference was found in intraoperative vein graft flow, postoperative bleeding, or leg wound infections (4% in each group) | 156 | 100 |
| 29 | Etiwy et al[39] | 2018 | Annals of Thoracic Surgery | To assess the time and cost required for suture fixation with the automated device versus conventional hand tying in sternotomy for mitral or tricuspid  ring annuloplasty | The time required to affix the annuloplasty device to the valve annulus | The times taken to affix a mitral annuloplasty band or ring were 6.1 ± 0.9 min for manual tying versus 3.1 ± 0.4 min for automated fasteners (p < 0.0001); when calculated per annuloplasty stitch, the values were 22 ± 2 s versus 12 ± 1.1 s, respectively (p < 0.0001) | 50 | 50 |
| 30 | Long et al[40] | 2018 | Annals of Thoracic Surgery | To confirm that VATS is not inferior or even superior to open operation for early stage NSCLC in terms of short-term and oncologic efficacy | 5-year overall and disease-free survival | Not reported (interim analysis) | 508 | 481 |
| 31 | Salim et al[41] | 2018 | Annals of Thoracic Surgery | To compare T2-4 versus T3-4 sympathectomy for the treatment of hyperhidrosis | Improvement of palm sweating, development of complications, patient satisfaction, and quality of life | Overdry hands were significant in group A (p=0.032). Compensatory sweating was significant in group A after the first month (p=0.016), after 6 months (p=0.022), and after 12 months (p=0.025). In group B, very satisfied patients were significant after 6 and 12 months (p=0.002 and 0.000, respectively) | NR | 120 |
| 32 | Halfwerk et al[42] | 2019 | Annals of Thoracic Surgery | To compare MiECC to an advanced standard system with respect to blood loss | Postoperative blood loss after 12 hours and at drain removal | MiECC patients (n=63) had a significant lower blood loss (230 mL, 95% confidence interval: 203 to 261 mL) than regular patients (n=62) after 12 hours (288 mL, 95% confidence interval: 241 to 344 mL, p=0.04) | NR | 180 |
| 33 | Jeong et al[43] | 2019 | Annals of Thoracic Surgery | To compare the early outcomes of a concomitant maze procedure using N2O-based cryoablation (the N2O group) versus argon gas-based cryoablation (the argon group) in patients with persistent atrial fibrillation (AF) | Normal sinus rhythm at 1 year | Normal sinus rhythm was maintained in 26 patients in the N2O group and 26 patients in the argon group (86.7% versus 86.7%, p=1.000) | 66 | 60 |
| 34 | Leshnower et al[44] | 2019 | Annals of Thoracic Surgery | To compare two different established neuroprotective strategies in patients undergoing elective transverse hemiarch replacement | Composite of stroke, transient ischemic attack, and magnetic resonance imaging–adjudicated injury | The primary end point was achieved in 100% of MHCA+ACP patients compared with 45% of DHCA+RCP patients (p < 0.01). | NR | 20 |
| 35 | ESVIL[45] | 2015 | JAMA Surgery | To determine whether Ivor-Lewis esophagectomy is associated with increased postoperative complications compared with the Sweet procedure | Operative morbidity (any surgical or nonsurgical complications) | Although there was no significant difference between the 2 groups regarding the incidence of each single complication, a significantly higher morbidity rate was found in the Sweet group (62 of 150 (41.3%)) than in the Ivor-Lewis group (45 of 150 (30%)) (P = .04) | 1224 | 300 |
| 36 | Damgaard et al[46] | 2008 | JTCVS | To compare outcomes in patients undergoing total arterial revascularization during coronary artery bypass grafting | Angiographic 1- and 5-year distal anastomotic patency and cardiac event-free survival at 1, 5, and 10 years postoperatively | Three months’ follow-up for the arterial versus conventional groups showed the following: deaths: 1 (0.6%) versus 0; stroke: 3 (1.9%) versus 3 (1.8%); myocardial infarction: 6 (3.7%) versus 4 (2.4%); sternal wound reoperation: 4 (2.5%) versus 0 (P=.054); arm and leg wound complications requiring hospitalization: 3 (1.9%) versus 6 (3.5%) (P=.50), respectively | 630 | 331 |
| 37 | Droghetti et al[47] | 2008 | JTCVS | To evaluate 2 different surgical techniques (staple versus collagen patch) for the completion of interlobar fissures during pulmonary lobectomy to establish which is superior in preventing air leakage | To assess the percentage of demonstrated intraoperative alveolar air leak effectively sealed after application of the patch (TachoSil) in ES and to compare the proportion of patients incthe experimental and control groups who were free of air leaks throughout hospitalization | Statistically significant reductions of air leakage were found in the ES group in the overall incidence of air leaks (50%vs 95%, P=.0001), duration of air leaks (1.7 days vs 4.5 days, P=.003), and procedure costs (425 euros vs 630.5 euros, P=.0001) | NR | 40 |
| 38 | Falk et al[48] | 2008 | JTCVS | To determine how preservation of leaflet structure in combination with premeasured neochordae compares with the widely adopted technique of leaflet resection | Valvular hemodynamics and orifice size (the postoperative MV orifice area of 0.5 cm2 or more and a difference in leaflet coaptation length of 1.5 mm or more) | Intraoperative transesophageal echocardiography showed a significantly longer line of mitral valve leaflet coaptation after implantation of loops (7.6±3.6 mm) than after resection (5.9±2.6 mm; P=.03). Early and mid-term echocardiographic follow-up revealed excellent valve function in the majority of patients, with no significant difference in mitral orifice area (3.6±1.0 cm^2^ vs 3.7±1.1 cm^2^, P=0.4) | 896 | 129 |
| 39 | Glineur et al[49] | 2008 | JTCVS | To compare the clinical, functional, and angiographic evolution of saphenous vein versus right gastroepiploic artery grafts at 6 months and 3 years | Major adverse cerebrocardiovascular events and proportion of grafts patent or functional at follow-up angiography | At follow-up there was no significant difference in major adverse cerebrocardiovascular events between the 2 groups. At the 6-month angiographic follow-up, 91% of the anastomoses in the right gastroepiploic artery group and 95% of the anastomoses in the saphenous vein graft group were controlled patent (P=.92) | 1397 | 244 |
| 40 | Guenzinger et al[50] | 2008 | JTCVS | To evaluate the impact of complete supraannular positioning of mechanical aortic bileaflet valves | Early hemodynamic and clinical performance of 2 mechanical aortic valve prostheses during rest and exercise, to analyze the impact of complete supraannular valve positioning, and to evaluate prosthesis-specific differences in valve sizing and valve size labeling | By grouping the data on the basis of a patient’s tissue annulus diameter, no significant difference of either valve was detected with regard to mean pressure gradient and effective orifice area index at rest | NR | 80 |
| 41 | Kamiya et al[51] | 2008 | JTCVS | To investigate the influence of skeletonized internal thoracic artery  harvesting on the sternal microcirculation in the perioperative phase | Oxygen saturation and blood flow at the presternal and retrosternal sides in the upper, middle, and lower parts | Skeletonization had no advantage in maintaining presternal microcirculation. Retrosternal microcirculation also deteriorated at all measurement points after internal thoracic artery harvesting in both groups. However, the deterioration of the retrosternal microcirculation was significantly less in group 1 at the middle and lower sternum | NR | 24 |
| 42 | Narang et al[52] | 2008 | JTCVS | To compare stentless and stented bioprostheses | Clinical outcomes, hemodynamic performance, and postoperative left ventricular mass regression | At 18±3 months postoperatively, the effective orifice area was greater in group A versus group B. Left ventricular ejection fraction, left ventricular mass index, functional class, and mean gradient were similar in patients of subgroup I (left ventricular ejection fraction .50%) from both groups | NR | 62 |
| 43 | EPIC[53] | 2009 | JTCVS | To evaluate the efficacy of the PAS-Port device which allows an automated proximal anastomosis to be performed without aortic clamping | Angiographic patency (<50% stenosis) 9 months after surgical intervention | The 9-month graft patency was 82.0% (150/183) for hand-sewn and 80.3% (147/183) for PAS-Port grafts. The patency rate of PAS-Port anastomoses was statistically noninferior to that of hand-sewn anastomoses (95% lower confidence limit for difference, -7.95%) | 589 | 220 |
| 44 | Fattouch et al[54] | 2009 | JTCVS | To evaluate the impact of the off-pump technique on clinical results | The incidence of in-hospital death and outcomes (low cardiac output syndrome, prolonged mechanical and pharmacologic cardiac support, prolonged mechanical ventilation support, and postoperative length of stay in intensive care unit and hospital) | In-hospital mortality was 7.7% (5 patients) in the on-pump group and 1.6% (1 patient) in the off-pump group (P=.04). Statistically significant differences were found between the 2 groups concerning the incidence of low cardiac output syndrome (P=.001), time of inotrope drugs support (P=.001), time of mechanical ventilation (P=.006), reoperation for bleeding (P=.04), intensive care unit stay (P= .01), and in-hospital stay (P=.02) | NR | 128 |
| 45 | Formica et al[55] | 2009 | JTCVS | To verify the systemic inflammatory response, inflammatory myocardial damage, and early clinical outcome in coronary surgery with the miniaturized extracorporeal circulation system or on the beating heart | To analyze 1) the myocardial damage: troponin T (TnT), myoglobin, total creatine kinase (CK), and CK mass 2) the inflammatory response: IL-6, TNF-α, C-reactive protein, leukocyte, neutrophil, and monocyte cell counts; 3) the hemodilution: hematocrit and hemoglobin; and 4) the coagulative disorder: fibrinogen and platelet count | Release of interleukin-6 was higher in the off-pump coronary artery bypass grafting group 24 hours after the operation (P=.03), whereas levels of tumor necrosis factor-α were not different in both groups. Cardiac release of interleukin-6, tumor necrosis factor-a, and blood lactate were not different in both groups. Release of troponin T was not significantly different in both groups. Levels of creatine kinase mass were statistically higher in the miniaturized extracorporeal circulation group than in the off-pump coronary artery bypass grafting group, but only at the end of the operation (P<.0001). Hemoglobin levels were significantly higher in the miniaturized extracorporeal circulation group than in the off-pump coronary artery bypass grafting group after 24 hours (P= .01) | NR | 60 |
| 46 | POINT[56] | 2009 | JTCVS | To evaluate the effect of adding mitral valve repair to coronary artery bypass grafting on clinical outcomes and left ventricular remodeling in patients who underwent coronary artery bypass grafting alone versus coronary artery bypass grafting plus mitral valve repair | To evaluate the effect of adding MVR to CABG on the clinical status of patients measured based on New York Heart Association (NYHA) functional class and on postoperative reversal of left ventricular remodeling measured based on left ventricular end-systolic diameter (LVESD), left ventricular end-diastolic diameter (LVEDD), and left ventricular ejection fraction (LVEF) | A significant difference was found between the 2 groups with regard to mean New York Heart Association class (P<.0001), left ventricular end-diastolic diameter (P<.01), left ventricular end-systolic diameter (P<.01), pulmonary arterial pressure (P<.0001), and left atrial size (P<.01) | 132 | 102 |
| 47 | Sakwa et al[57] | 2009 | JTCVS | To determine differences in blood loss and transfusion associated with a minimized cardiopulmonary bypass circuit versus a standard bypass circuit | Differences in blood loss and transfusion | Hematocrit, equivalent at baseline, was higher in minimized circuit cohort at lowest point during cardiopulmonary bypass (31.5%±3.9% vs. 25.5%±3.7%), after protamine (31.6%±3.9% vs 29.2%±3.7%), and on intensive care unit arrival (35.2%±4.1% vs 31.8%±3.5%, P<.001). Fewer red blood cells (148 vs 19 units) were given in minimized circuit group (P<.0001) | NR | 199 |
| 48 | Stand in Y Mammary[58] | 2009 | JTCVS | To determine if the use of 2 arterial conduits rather than a single conduit in multivessel coronary artery bypass grafting significantly improves results despite the concomitant use of saphenous vein grafts and whether any among different configurations of composite grafts (left/right thoracic arteries and radial artery) offers an advantage over the others | In-hospital outcomes (mortality rate and morbidity), 2-year freedom from all-cause death, and adverse cardiac event–free survival (adverse cardiac events included cardiac death, acute myocardial infarction, recurrent angina, graft occlusion at coronary angiography, redo coronary surgery, or percutaneous transluminal coronary angioplasty) | The rate of cerebrovascular complications was not statistically lower among patients receiving 2 arterial grafts. At 2 years, overall survival was not significantly different among groups (P=.59). Cardiac event–free survival was significantly better in patients receiving 2 arterial grafts versus control subjects (P<.0001), even among elderly patients (P=.022). The 3 investigated strategies using 2 arterial conduits were similar concerning early and midterm results | 1769 | 815 |
| 49 | Belcher et al[59] | 2010 | JTCVS | To compare BioGlue and Vivostat in the control of postoperative air leak | Duration of air leak, time to intercostal drain removal, and length of hospital stay | Median duration of air leak was 3 (0–32) days versus 2 (0–33) days for patients who received BioGlue and Vivostat, respectively (P=.677). Time to intercostal drain removal was 5 (1–32) days in the BioGlue group compared with 5 (1–34) days for the Vivostat group (P=.473). Median hospital stay was 8 (3–22) days versus 7 (2–29) days for the BioGlue and Vivostat groups, respectively (P=.382) | 378 | 103 |
| 50 | Markman et al[60] | 2010 | JTCVS | To evaluate the incidence and severity of pain by using skeletonized internal thoracic artery harvesting rather than pedicled harvesting | The area of harvest dysesthesia, expressed as a percentage of the hemithoracic area (7 weeks, 21 weeks) | The incidence of harvest dysesthesia at 7 weeks was 14% in the skeletonized group versus 50% in the pedicled group (P=.02). These differences were not sustained at 21 weeks, as the median area of harvest dysesthesia in both groups was 0% (P=.89) and the incidence was 24% and 25% in the skeletonized and pedicled groups, respectively (P=1.0) | NR | 41 |
| 51 | Ponce Gonzalez et al[61] | 2010 | JTCVS | To compare the long-term effects of conventional and simplified thoracic sympathectomy on cardiopulmonary function | Forced spirometry, body plethysmography, measurement of the diffusing capacity of the lung for carbon monoxide (DLCO), and exercise tests at 1 year | No significant differences were found between the conventional and simplified thoracic sympathectomy groups | NR | 32 |
| 52 | Roshanali et al[62] | 2010 | JTCVS | To lower the failure rate of tricuspid repair (TR) for functional tricuspid regurgitation by reducing leaflet tethering via pericardial patch augmentation when the preoperative probability of recurrence was high | Failure rate of TR (residual tricuspid regurgitation in tricuspid valve augmentation) | Postoperative tricuspid regurgitation was different between the groups (P<.05): 16.0%and 28.0%of patients in the De Vega group, 8.0%and 14.0%of patients in the ring annuloplasty group, 4.0%and 10.0%of patients in the De Vega pericardial patch augmentation group, and 2.0% and 8.0% of patients in the ring annuloplasty pericardial patch augmentation group had postoperative tricuspid regurgitation at 1-month and 1-year follow-up, respectively | NR | 210 |
| 53 | Salehi Omran et al[63] | 2010 | JTCVS | To assess the prophylactic effect of ventral cardiac denervation on reducing atrial fibrillation after coronary artery bypass grafting | Atrial fibrillation incidence | Atrial fibrillation incidence was significantly different between the groups (P=.025), with an incidence of 20.9%in the ventral cardiac denervation group and 10% in the control group. Atrial fibrillation occurred in 34 of the 220 patients, and ventral cardiac denervation was considered as a variable to evaluate its possible role in the prevention of postoperative atrial fibrillation | NR | 220 |
| 54 | Wiklund et al[64] | 2010 | JTCVS | To evaluate the clinical and angiographic outcomes of a second-generation anastomotic device used for saphenous vein grafts | 1-year patency for vein grafts anastomoses | The 1-year patency rate for study grafts constructed with the anastomotic connector was 92.2% (118/128) and for hand-sutured grafts, 91.7% (121/132) | NR | 151 |
| 55 | ACSOG Z0030[65] | 2011 | JTCVS | To determine whether mediastinal lymph node dissection improves survival compared with mediastinal  lymph node sampling in patients undergoing resection for N0 or non-hilar N1, T1, or T2 non–small cell lung cancer | Overall survival (5 years) | The median survival is 8.1 years for mediastinal lymph node sampling and 8.5 years for mediastinal lymph node dissection (P=.25). The 5-year disease-free survival was 69%(95%confidence interval, 64–74) in the mediastinal lymph node sampling group and 68%(95% confidence interval, 64–73) years in the mediastinal lymph node dissection group (P=.92) | NR | 1111 |
| 56 | Al-Rashidi et al[66] | 2011 | JTCVS | To compare the effectiveness, time required for de-airing, and safety of a newly developed de-airing technique for open left heart surgery (Lund technique) with a standardized carbon dioxide insufflation technique | The severity of gas emboli observed on transesophageal echocardiography and the number of microembolic signals recorded by transcranial Doppler | The severity of gas emboli observed on transesophageal echocardiography and the number of microembolic signals recorded by transcranial Doppler were significantly lower in the Lund group during the de-airing procedure (P = .00634) and in the first 10 minutes after weaning from cardiopulmonary bypass (P = .000377) | NR | 20 |
| 57 | Bonacchi et al[67] | 2011 | JTCVS | To evaluate the ꭕ-configuration, a new cannulation strategy for VV-ECMO | Efficacy of blood oxygenation was obtained by gas–blood analysis, by blood samples obtained at arterial, central venous, and pulmonary artery lines, and by ECMO inflow and outflow lines | In group NS, on-ECMO time, post-ECMO mechanical ventilation time, and ECMO overall results were significantly better than in group C. During high-flow VV-ECMO, pulmonary and systemic arterial oxygen saturation and arterial oxygen tension were significantly higher in group NS, and blood recirculation fraction was significantly lower | NR | 30 |
| 58 | De Leyn et al[68] | 2011 | JTCVS | To evaluate the efficacy and safety of a synthetic bioresorbable pleural sealant to treat air leaks after pulmonary resection | Percentage of patients remaining air leak free until discharge | The overall success rates for intraoperative air leak sealing were as follows: sealant group, 71.0%; control group, 23.7% (P<.001). For grade 2 and 3 air leaks (n=77), the intraoperative sealing rates were as follows: sealant group, 71.7%; control group, 9.1% (P<.001). More patients with grade 2 and 3 air leaks had their leaks remain sealed in the sealant group (43.5%vs 15.2%, P=.013). The median time from skin closure to last observable air leak was 6 hours (sealant group) versus 42  hours (control group, P=.718) | 136 | 121 |
| 59 | Michaux et al[69] | 2011 | JTCVS | To determine if right ventricular global and overall systolic functions are better preserved 3 months after  off-pump surgery than after conventional coronary bypass surgery | The echocardiographic indicators of RV global and overall systolic function | There were no significant intergroup differences in any echocardiographic marker of right ventricular function | NR | 50 |
| 60 | Speziale et al[70] | 2011 | JTCVS | To determine if the results of mitral repair for complex Barlow valves are adequate and support earlier intervention | No primary outcome excplicityly defined | Not reported | 618 | 140 |
| 61 | Benedetto et al[71] | 2012 | JTCVS | To assess if concomitant tricuspid valve annuloplasty in patients  with tricuspid annulus dilatation (≥40 mm) prevents tricuspid regurgitation progression after mitral valve surgery | The occurrence of moderate to severe (≥3+) functional TR at 1 year follow-up | At 12 months follow-up, tricuspid regurgitation was absent in 71% (n=15) versus 19% (n=4) of patients in the treatment and control groups, respectively (P=.001) | NR | 44 |
| 62 | Chaudhuri et al[72] | 2012 | JTCVS | To analyze neurocognitive outcomes of patients after open-chamber cardiac surgery to determine whether carbon dioxide pericardial insufflation reduces incidence of neurocognitive decline (primary end point) as measured 6 weeks postoperatively and to assess the utility of carbon dioxide insufflation in cardiac chamber deairing as assessed by transesophageal echocardiography | Incidence of neurocognitive decline as measured 6 weeks postoperatively | Neurocognitive testing showed no clinically significant differences in z scores between preoperative and postoperative testing | 146 | 125 |
| 63 | On-off[73] | 2012 | JTCVS | To analyze the risk reduction of cardiopulmonary bypass complications between on-pump and off-pump coronary artery bypass grafting in high-risk patients | Composite of mortality and major complications comprising myocardial infarction (MI), neurologic complications, renal failure, adult respiratory distress syndrome (ARDS), and reoperation for bleeding, occurring within 30 days after surgery | According to the intention to treat analysis, the rate of the composite primary end point was significantly lower (unadjusted P=.009, adjusted P=.010) in the off-pump group (5.8% vs 13.3%). The risk of experiencing the primary end point was significantly greater for the on-pump group (unadjusted odds ratio, 2.51; 95% confidence interval, 1.23–5.10; P=.011; adjusted odds ratio, 3.07; 95% confidence interval, 1.32–7.14; P=.009). | NR | 411 |
| 64 | SAVE RITA[74] | 2012 | JTCVS | To compare early angiographic  patency rates and clinical outcomes between the saphenous vein and right interval thoracic artery as a Y-composite graft | The 1-year angiographic patency rate of the distal anastomoses performed with a side-arm composite graft | Early angiography demonstrated an overall patency rate of 99.4% (771 of 776 distal anastomoses). Patency rates of the side-arm Y-composite graft (saphenous vein vs right internal thoracic artery) were 98.8% (245 of 248) and 99.5% (207 of 208) in the saphenous vein and right internal thoracic artery groups, respectively (P=.629) | 496 | 224 |
| 65 | Suri et al[75] | 2012 | JTCVS | To determine whether there are clinically important early differences among the Edwards Magna, Sorin Mitroflow, or St. Jude Epic bioprostheses | Early hemodynamic performance by echocardiography | Postoperative echocardiography showed small but statistically significant differences overall between the Magna, Mitroflow, and Epic valves in mean gradient (14.2 mm Hg, 16.3 mm Hg, 16.5 mm Hg, respectively; P=.011), aortic valve area (2.05 cm^2^, 1.88 cm^2^, 1.86 cm^2^, respectively; P=.012), and indexed aortic valve area (1.05 cm^2^/m^2^, 0.97 cm^2^/m^2^, 0.95 cm^2^/m^2^, respectively; P=.012) | NR | 300 |
| 66 | STET[76] | 2013 | JTCVS | To compare off-pump coronary artery bypass surgery carried out via a left anterolateral thoracotomy (ThoraCAB) or via a conventional median sternotomy (OPCAB) | The time from surgery to fitness for hospital discharge as defined by objective criteria | The median time from surgery to fitness for discharge was 6 days (interquartile range, 4-7) in the ThoraCAB group versus 5 days (interquartile range, 4-7) in the OPCAB group (P=.53) | 465 | 93 |
| 67 | Bouchard et al[77] | 2014 | JTCVS | To assess the effect of treating FIMR with annuloplasty when mitral  regurgitation is moderate | The LV dimension changes at 1 year | The left ventricular ejection fraction was significantly better at 3 months in the CABG alone group, although at 12 months, the left ventricular ejection fraction in the 2 groups had improved similarly | NR | 67 |
| 68 | Svensson et al[78] | 2015 | JTCVS | To perform a randomized trial of brain protection during total aortic arch replacement and identify the best way to assess brain injury | Composite of (1) hospital death from neurologic causes, (2) postoperative clinical stroke, (3) brain imaging changes, and (4) reliable neurocognitive decline | The primary study composite neurologic end point, obtained by 10-fold multiple imputation of individual components, occurred in 22 of the 60 patients undergoing RBP and 15 of the 61 patients undergoing ABP (P=.2) | NR | 121 |
| 69 | Lee et al[79] | 2016 | JTCVS | To evaluate 3 surgical techniques for left atrial appendage exclusion with long-term follow up to define effectiveness | Success of closure (assessed in real time in multiple views after cessation of cardiopulmonary bypass by an experienced echocardiographer), including no remnant pouch >1 cm in maximum length after closure (stump) and the absence of a color flow jet between the left atrium (LA) and the LAA (gap) | In late follow-up, 1 of 7 patients in the IL group (14%) had a stump, compared with 2 of 8 (25%) in the StEx group and 3 of 6 (50%) in the SxEx group (P=.35). The overall failure rate was 57%: 5 of 8 (63%) in the IL group, 6 of 10 (60%) in the StEx group, and 5 of 10 (50%) in the SxEx group (P=.85). No patient had a stroke at any time during follow-up | 214 | 28 |
| 70 | Allen et al[80] | 2017 | JTCVS | To evaluate sternal healing, complications, and costs after sternotomy closure with rigid plate fixation or wire cerclage | Sternal healing based on CT evaluation by an independent core laboratory using a validated method at 6 months | Rigid plate fixation resulted in better sternal healing scores at 3 (2.6±1.1 vs 1.8±1.0; P<.0001) and 6 months (3.8±1.0 vs 3.3±1.1; P=.0007) and greater sternal union rates at 3 (41% (42/103) vs 16% (16/102); P<.0001) and 6 months (80% (81/101) vs 67% (67/100); P=.03) compared with wire cerclage | 461 | 236 |
| 71 | Halkos et al[81] | 2017 | JTCVS | To determine the impact of different aortic clamping strategies on the incidence of cerebral embolic events during coronary artery bypass grafting (CABG) | The number of HITS detected during TCD ultrasonography | In the off-pump group, the median number of total HITS were higher in the CFD subgroup (30.0; interquartile range (IQR), 22-43) compared with the partial clamp subgroup (7.0; IQR, 0-16; P<.0001). In the CFD subgroup, the median number of total HITS was significantly lower for patients with 1 CFD compared with patients with>1 CFD (12.5 (IQR, 4-19) vs 36.0 (IQR, 25-47); P=.001) | 1235 | 142 |
| 72 | Ad et al[82] | 2018 | JTCVS | To examine whether expanding del Nido cardioplegia to adult cardiac surgery confers benefits in surgical workflow and clinical outcome compared with blood-based cardioplegia | Myocardial preservation by return to spontaneous rhythm; defibrillation requirement; inotropes; and troponin levels at 4 time points: baseline (at anesthesia induction), 2 hours after termination of CPB, 12 hours after admission to a cardiovascular intensive care unit (CVICU), and 24 hours after admission to a CVICU | There was no significant difference on CPB time (97 vs 103 minutes; P=.288) or crossclamp time (70 vs 83 minutes; P=.018). The del Nido group showed higher return to spontaneous rhythm (97.7% vs 81.6%; P=.023) and fewer patients required inotropic support (65.1% vs 84.2%; P=.050), but did not reach statistical significance. For del Nido group patients, troponin levels did not increase as much as for control patients (P=.040), but statistical significance was not reached | NR | 89 |
| 73 | Marasco et al[83] | 2018 | JTCVS | To evaluate if biocompatible plastic cable ties would achieve a more rigid sternal fixation, reducing postoperative pain and analgesia requirements | Pain and analgesia requirements in the early postoperative period | There were no significant differences between groups in postoperative pain, analgesia, or early ventilator requirements. Patients in the ZIPFIX group had significantly more movement in the sternum and manubrium on ultrasound at 4 weeks | NR | 120 |
| 74 | Mini-Stern[84] | 2018 | JTCVS | To establish whether mini-sternotomy leads to quicker postoperative recovery and shorter hospital stay after first-time isolated AVR | Duration of postoperative hospital stay and the time to fitness for discharge from hospital after AVR | Compared with the FS group, the MS group had a longer hospital length of stay (mean, 9.5 days vs 8.6 days) and took longer to achieve fitness for discharge home (mean, 8.5 days vs 7.5 days) | 1024 | 222 |
| 75 | Wang et al[85] | 2018 | JTCVS | To evaluate the safety and efficacy of the addition of the cut-and-sew  Maze III procedure (CSM) for mitral valve replacement (MVR) in patients with atrial fibrillation (AF) associated with rheumatic mitral valve disease (RMVD) | Composite of freedom from stroke and death at 1 year | One-year freedom from stroke or death was better in the Maze III group compared with the non-Maze group (P=.0028; hazard ratio, 0.2653; 95% confidence interval, 0.1122 to 0.6270). The risk of AF recurrence in the Maze III group was 0.002-fold that in non-Maze group (P=.000) | 180 | 130 |
| 76 | Braathen et al[86] | 2019 | JTCVS | To compare hemodynamic profiles of the Trifecta to our standard Mosaic  Ultra biological valve | Mean and maximum gradients and EOAI measured 6 months postoperatively with transthoracic echocardiography | There were lower transvalvular gradients in the Trifecta compared with the Mosaic Ultra group for the given annulus sizes | NR | 90 |
| 77 | CANON[87] | 2019 | JTCVS | To determine whether using any of the 2 selected modifications of OPCAB could decrease the incidence of neuropsychiatric complications | Incidence of postoperative delirium (PD) and early postoperative cognitive dysfunction (ePOCD) | The incidence of PD was 35.9% in the control (OPCAB) arm, 32.8% in the CO2FF arm, and 12.5%in the ANA arm (x^2^ [2, N=191] = 10.17; P=.006). Post hoc tests revealed that the incidence of PD in the ANA arm differed from thatcin the OPCAB arm (odds ratio [OR], 0.26; 95% confidence interval [CI], 0.09-0.68; P=.002). The incidence of ePOCD was 34.4% in the OPCAB arm, 28.1% in the CO2FF arm, and 9.5% in the ANA arm (x^2^ [2, N=191] = 11.58; P=.003) | 269 | 192 |
| 78 | Jiwnani et al[88] | 2019 | JTCVS | To evaluate if a modified technique of posterolateral thoracotomy and  closure, preserving the intercostal neurovascular bundle, would reduce acute and chronic post-thoracotomy pain | Worst postoperative pain score in the first 3 postoperative days | There was no difference seen in the worst (mean) postoperative pain scores (3.71 vs 3.83, difference 0.12; 99% confidence interval [CI], -0.7 to +0.9; P=0.7) | 340 | 90 |
| 79 | Suzuki et al[89] | 2019 | JTCVS | To confirm the noninferiority of segmentectomy to lobectomy in regard to prognosis | Overall survival | No mortality was noted | 1319 | 1106 |
| 80 | HeartMate II[90] | 2009 | NEJM | To determine the efficacy of a new continuous-flow left ventricular assist device with a pulsatile device | Composite of survival free from disabling stroke and reoperation to repair or replace the device at 2 years | The primary composite end point was achieved in more patients with continuous-flow devices than with pulsatile-flow devices (62 of 134 (46%) vs. 7 of 66 (11%); P<0.001; hazard ratio, 0.38; 95% confidence interval, 0.27 to 0.54; P<0.001), and patients with continuous- flow devices had superior actuarial survival rates at 2 years (58% vs. 24%, P = 0.008) | NR | 200 |
| 81 | ROOBY[91] | 2009 | NEJM | To determine if coronary artery bypass grafting without cardiopulmonary bypass (off-pump CABG) reduces the number of complications related to the heart–lung machine | Composite of death from any cause, a repeat revascularization procedure, or a nonfatal myocardial infarction within 1 year after surgery | There was no significant difference between off-pump and on-pump CABG in the rate of the 30-day composite outcome (7.0% and 5.6%, respectively; P = 0.19). The rate of the 1-year composite outcome was higher for off-pump than for on-pump CABG (9.9% vs. 7.4%, P = 0.04) | 9663 | 2203 |
| 82 | STICH[92] | 2009 | NEJM | To address whether surgical ventricular reconstruction added to coronary-artery bypass grafting (CABG) would decrease the rate of death or hospitalization for cardiac causes, as compared with CABG alone | Composite of death from any cause and hospitalization for cardiac causes | No significant difference was observed in the primary outcome, which occurred in 292 patients (59%) who were assigned to undergo CABG alone and in 289 patients (58%) who were assigned to undergo CABG with surgical ventricular reconstruction (hazard ratio for the combined approach, 0.99; 95% confidence interval, 0.84 to 1.17; P = 0.90) | NR | 1000 |
| 83 | CORONARY[93] | 2012 | NEJM | To establish the benefits and risk of performing coronary-artery bypass grafting (CABG) with a beating-heart technique (off-pump CABG), as compared with cardiopulmonary  bypass (on-pump CABG) | The first coprimary outcome was a composite of death, nonfatal stroke, nonfatal myocardial infarction, or new renal failure requiring dialysis at 30 days after randomization. The second coprimary outcome was the first coprimary outcome plus repeat coronary revascularization at a mean of 5 years | There was no significant difference in the rate of the primary composite outcome between off-pump and on-pump CABG (9.8% vs. 10.3%; hazard ratio for the off pump group, 0.95; 95% confidence interval (CI), 0.79 to 1.14; P = 0.59) or in any of its individual components | NR | 4752 |
| 84 | GOPCABE[94] | 2013 | NEJM | To investigate the benefits of coronary artery bypass grafting without cardiopulmonary bypass in elderly patients | Composite of death, stroke, myocardial infarction, repeat revascularization, or new renal-replacement therapy at 30 days and at 12 months after surgery | At 30 days after surgery, there was no significant difference between patients who underwent off-pump surgery and those who underwent on-pump surgery in terms of the composite outcome (7.8% vs. 8.2%; odds ratio, 0.95; 95% confidence interval (CI), 0.71 to 1.28; P = 0.74) or four of the components (death, stroke, myocardial infarction, or new renal replacement therapy) | 4355 | 2403 |
| 85 | Acker et al[95] | 2014 | NEJM | To evaluate the efficacy and safety of mitral valve repair versus chordal sparing replacement for the treatment of ischemic mitral regurgitation | Left ventricular end-systolic volume index (LVESVI) at 12 months | At 12 months, the mean LVESVI among surviving patients was 54.6±25.0 ml per square meter of body-surface area in the repair group and 60.7±31.5 ml per square meter in the replacement group (mean change from baseline, −6.6 and −6.8 ml per square meter, respectively). The rate of death was 14.3% in the repair group and 17.6% in the replacement group (hazard ratio with repair, 0.79; 95% confidence interval, 0.42 to 1.47; P = 0.45 by the log-rank test). There was no significant between-group difference in LVESVI after adjustment for death (z score, 1.33; P = 0.18) | 3458 | 251 |
| 86 | Smith et al[96] | 2014 | NEJM | To determine the benefits of adding mitral valve repair to coronary artery bypass in patients with ischemic mitral regurgitation | Left ventricular end-systolic volume index (LVESVI), a measure of left ventricular remodeling, at 1 year | The mean LVESVI among surviving patients was 46.1±22.4 ml per square meter of body-surface area in the CABG-alone group and 49.6±31.5 ml per square meter in the combined-procedure group (mean change from baseline, −9.4 and −9.3 ml per square meter, respectively) | 725 | 301 |
| 87 | Gillinov et al[97] | 2015 | NEJM | To evaluate the safety and effectiveness of surgical ablation of atrial fibrillation in patients undergoing mitral valve surgery | Freedom from atrial fibrillation at both 6 months and 12 months (as assessed by means of 3-day Holter monitoring) | There was no significant difference in the rate of freedom from atrial fibrillation between patients who underwent pulmonary-vein isolation and those who underwent the biatrial maze procedure (61.0% and 66.0%, respectively; P = 0.60) | 3502 | 260 |
| 88 | ENDURANCE[98] | 2017 | NEJM | To compare a newer LVAD design (a small intrapericardial centrifugal-flow device) against existing  technology (a commercially available axial-flow device) in patients with advanced heart failure who were ineligible for heart transplantation | Survival at 2 years free from disabling stroke or device removal for malfunction or failure | The primary end point was achieved in 164 patients in the study group and 85 patients in the control group. The analysis of the primary end point showed noninferiority of the study device relative to the control device (estimated success rates, 55.4% and 59.1%, respectively, calculated by the Weibull model; absolute difference, 3.7 percentage points;95% upper confidence limit, 12.56 percentage points; P = 0.01 for noninferiority). | 559 | 445 |
| 89 | MOMENTUM 3[99] | 2017 | NEJM | To investigate the effects of a new magnetically levitated centrifugal  continuous-flow pump that was engineered to avert thrombosis | Composite of survival free of disabling stroke (with disabling stroke indicated by a modified Rankin score >3; scores range from 0 to 6, with higher scores indicating  more severe disability) or survival free of reoperation to replace or remove the device at 6 months after implantation | The primary end point occurred in 131 patients (86.2%) in the centrifugal-flow pump group and in 109 (76.8%) in the axial-flow pump group (absolute difference, 9.4 percentage points; 95% lower confidence boundary, −2.1 P<0.001 for noninferiority; hazard ratio, 0.55; 95% confidence interval (CI), 0.32 to 0.95 (two-tailed P = 0.04 for superiority)) | NR | 294 |
| 90 | FREGAT[100] | 2019 | NEJM | To determine whether hybrid minimally invasive esophagectomy results in lower morbidity than open  esophagectomy | Intraoperative or postoperative complication of grade II or higher according to the Clavien–Dindo classification (indicating major complication leading to intervention) within 30 days | A total of 37 patients (36%) in the hybrid procedure group had a major intraoperative or postoperative complication, as compared with 67 (64%) in the open-procedure group (odds ratio, 0.31; 95% confidence interval [CI], 0.18 to 0.55; P<0.001) | 219 | 207 |
| 91 | El-Hamamsy et al[101] | 2010 | The Lancet | To compare long term outcomes after autograft aortic root replacement (Ross procedure) versus homograft aortic root replacement in adults | Overall survival at 10 years | At 10 years, four patients died in the autograft group versus 15 in the homograft group. Actuarial survival at 10 years was 97% (SD 2) in the autograft group versus 83% (4) in the homograft group. Hazard ratio for death in the homograft group was 4.61 (95% CI 1.71–16.03; p=0.0060) | NR | 216 |
| 92 | Biere et al[102] | 2012 | The Lancet | To assess whether minimally invasive oesophagectomy reduces morbidity compared with open esophagectomy | Pulmonary infection within the first 2 weeks after surgery and during the whole stay in hospital | 16 (29%) patients in the open oesophagectomy group had pulmonary infection in the first 2 weeks compared with five (9%) in the minimally invasive group (relative risk (RR) 0.30, 95% CI 0.12–0.76; p=0.005). 19 (34%) patients in the open oesophagectomy group had pulmonary infection in-hospital compared with seven (12%) in the minimally invasive group (0.35, 0.16–0.78; p=0.005) | 144 | 115 |
| 93 | MesoVATS[103] | 2014 | The Lancet | To compare efficacy in terms of overall survival, and cost, of VAT-PP and talc pleurodesis in patients with malignant pleural mesothelioma | Overall survival at 1 year | Overall survival at 1 year was 52% (95% CI 41–62) in the VAT-PP group and 57% (46–66) in the talc pleurodesis group (hazard ratio 1.04 (95% CI 0.76–1.42); p=0.81) | NR | 175 |

**Supplementary Table 4**: Trial reporting and citations

| Variable | No. (%) |
| --- | --- |
| Trials with a favorable outcome | 53 (56.9) |
| Trials with a neutral outcome^a^ | 37 (39.7) |
| Multiplicity | 53 (56.9) |
| - Multiple treatment groups | 5 (9.4) |
| - Multiple outcomes | 23 (43.4) |
| - Multiple analyses of the same outcome | 12 (22.6) |
| - Multiple outcomes + Multiple analyses of the same outcome | 11(20.8) |
| - Multiple treatment groups + Multiple outcomes | 1 (1.9) |
| - Multiple treatment groups + Multiple analyses of the same outcome | 1 (1.9) |
| Adjusted for multiple comparisons | 7 (13.2) |
| - Bonferroni’s adjustment | 6 (85.7) |
| - Modified alpha value | 1 (14.3) |
| Spin present | 9/53 (17.0) |
| Extent of spin | - |
| - None | 44 (83.0) |
| - In conclusion only | 4 (44.5) |
| - In 2 sections | 3 (33.3) |
| - In all sections | 2 (22.2) |
| Number of citations, median (IQR) | 25 (10-56) |

Abbreviations: IQR= interquartile range

Footnote: ^a^In three trials the primary outcome was not reported and therefore could not be classified as either neutral or favorable

**Supplementary Table 5**: Citation rate by journal

| Journal | No. of trials | Median number of citations |
| --- | --- | --- |
| *The Journal of Thoracic and Cardiovascular Surgery* | 44 | 20 [9-35] |
| *Annals of Thoracic Surgery* | 32 | 16 [7-40] |
| *The New England Journal of Medicine* | 10 | 347 [223-406] |
| *The Lancet* | 3 | 152 [117-406] |
| *Annals of Surgery* | 2 | 19 [10-27] |
| *JAMA Surgery* | 2 | 32 [23-41] |

Abbreviations: JAMA= Journal of the American Medical Association

**Supplementary Figure 1**: Number of Trials by Year (p-for-trend 0.07)

References:

[1] Thorpe KE, Zwarenstein M, Oxman AD, Treweek S, Furberg CD, Altman DG, et al. A pragmatic-explanatory continuum indicator summary (PRECIS): a tool to help trial designers. J Clin Epidemiol 2009;62:464–75. https://doi.org/10.1016/j.jclinepi.2008.12.011.

[2] Sepehrvand N, Alemayehu W, Das D, Gupta AK, Gouda P, Ghimire A, et al. Trends in the Explanatory or Pragmatic Nature of Cardiovascular Clinical Trials Over 2 Decades. JAMA Cardiol 2019;4:1122. https://doi.org/10.1001/jamacardio.2019.3604.

[3] Sepehrvand N, Alemayehu W, Das D, Gupta AK, Gouda P, Ghimire A, et al. Trends in the Explanatory or Pragmatic Nature of Cardiovascular Clinical Trials Over 2 Decades. JAMA Cardiol 2019. https://doi.org/10.1001/jamacardio.2019.3604.

[4] Flacco ME, Manzoli L, Boccia S, Capasso L, Aleksovska K, Rosso A, et al. Head-to-head randomized trials are mostly industry sponsored and almost always favor the industry sponsor. J Clin Epidemiol 2015;68:811–20. https://doi.org/10.1016/j.jclinepi.2014.12.016.

[5] Boutron I, Dutton S, Ravaud P, Altman DG. Reporting and interpretation of randomized controlled trials with statistically nonsignificant results for primary outcomes. JAMA 2010;303:2058–64. https://doi.org/10.1001/jama.2010.651.

[6] Li G, Taljaard M, Van den Heuvel ER, Levine MAH, Cook DJ, Wells GA, et al. An introduction to multiplicity issues in clinical trials: the what, why, when and how. Int J Epidemiol 2016:dyw320. https://doi.org/10.1093/ije/dyw320.

[7] Chan A-W, Hróbjartsson A, Haahr MT, Gøtzsche PC, Altman DG. Empirical evidence for selective reporting of outcomes in randomized trials: comparison of protocols to published articles. JAMA 2004;291:2457–65. https://doi.org/10.1001/jama.291.20.2457.

[8] Chen T, Li C, Qin R, Wang Y, Yu D, Dodd J, et al. Comparison of Clinical Trial Changes in Primary Outcome and Reported Intervention Effect Size Between Trial Registration and Publication. JAMA Netw Open 2019;2:e197242. https://doi.org/10.1001/jamanetworkopen.2019.7242.

[9] Mazzinari G, Ball L, Serpa Neto A, Errando CL, Dondorp AM, Bos LD, et al. The fragility of statistically significant findings in randomised controlled anaesthesiology trials: systematic review of the medical literature. Br J Anaesth 2018;120:935–41. https://doi.org/10.1016/j.bja.2018.01.012.

[10] Sterne JAC, Savović J, Page MJ, Elbers RG, Blencowe NS, Boutron I, et al. RoB 2: a revised tool for assessing risk of bias in randomised trials. BMJ 2019:l4898. https://doi.org/10.1136/bmj.l4898.

[11] Chen J-S, Hsu H-H, Huang P-M, Kuo S-W, Lin M-W, Chang C-C, et al. Thoracoscopic Pleurodesis for Primary Spontaneous Pneumothorax With High Recurrence Risk: A Prospective Randomized Trial. Ann Surg 2012;255:440–5. https://doi.org/10.1097/SLA.0b013e31824723f4.

[12] Alifano M, Jayle C, Bertin F, Magdeleinat P, Castier Y, Tiffet O, et al. Medical and Economic Evaluation of FOREseal Bioabsorbable Reinforcement Sleeves Compared With Current Standard of Care for Reducing Air Leakage Duration After Lung Resection for Malignancy: A Randomized Trial. Ann Surg 2017;265:45–53. https://doi.org/10.1097/SLA.0000000000001687.

[13] Cerfolio RJ, Bryant AS, Maniscalco LM. A Nondivided Intercostal Muscle Flap Further Reduces Pain of Thoracotomy: A Prospective Randomized Trial. Ann Thorac Surg 2008;85:1901–7. https://doi.org/10.1016/j.athoracsur.2008.01.041.

[14] Li X, Tu Y-R, Lin M, Lai F-C, Chen J-F, Dai Z-J. Endoscopic Thoracic Sympathectomy for Palmar Hyperhidrosis: A Randomized Control Trial Comparing T3 and T2-4 Ablation. Ann Thorac Surg 2008;85:1747–51. https://doi.org/10.1016/j.athoracsur.2008.01.060.

[15] Schimmer C, Reents W, Berneder S, Eigel P, Sezer O, Scheld H, et al. Prevention of Sternal Dehiscence and Infection in High-Risk Patients: A Prospective Randomized Multicenter Trial. Ann Thorac Surg 2008;86:1897–904. https://doi.org/10.1016/j.athoracsur.2008.08.071.

[16] Krishnamoorthy B, Najam O, Khan UA, Waterworth P, Fildes JE, Yonan N. Randomized Prospective Study Comparing Conventional Subcuticular Skin Closure With Dermabond Skin Glue After Saphenous Vein Harvesting. Ann Thorac Surg 2009;88:1445–9. https://doi.org/10.1016/j.athoracsur.2009.06.047.

[17] Suri RM, Zehr KJ, Sundt TM, Dearani JA, Daly RC, Oh JK, et al. Left Ventricular Mass Regression After Porcine Versus Bovine Aortic Valve Replacement: A Randomized Comparison. Ann Thorac Surg 2009;88:1232–7. https://doi.org/10.1016/j.athoracsur.2009.04.128.

[18] Allama AM. Intercostal Muscle Flap for Decreasing Pain After Thoracotomy: A Prospective Randomized Trial. Ann Thorac Surg 2010;89:195–9. https://doi.org/10.1016/j.athoracsur.2009.07.094.

[19] Yousefnia MA, Dehestani A, Saidi B, Roshanali F, Mandegar MH, Alaeddini F. Papillary Muscle Repositioning in Valve Replacement for Left Ventricular Dysfunction: Ischemic Mitral Regurgitation. Ann Thorac Surg 2010;90:497–502. https://doi.org/10.1016/j.athoracsur.2010.04.060.

[20] Aykut K, Celik B, Acıkel U. Figure-of-Eight Versus Prophylactic Sternal Weave Closure of Median Sternotomy in Diabetic Obese Patients Undergoing Coronary Artery Bypass Grafting. Ann Thorac Surg 2011;92:638–41. https://doi.org/10.1016/j.athoracsur.2011.04.034.

[21] Baumgartner FJ, Reyes M, Sarkisyan GG, Iglesias A, Reyes E. Thoracoscopic Sympathicotomy for Disabling Palmar Hyperhidrosis: A Prospective Randomized Comparison Between Two Levels. Ann Thorac Surg 2011;92:2015–9. https://doi.org/10.1016/j.athoracsur.2011.07.083.

[22] Lai F-C, Chen L, Tu Y-R, Lin M, Li X. Prevention of Chylothorax Complicating Extensive Esophageal Resection by Mass Ligation of Thoracic Duct: A Random Control Study. Ann Thorac Surg 2011;91:1770–4. https://doi.org/10.1016/j.athoracsur.2011.02.070.

[23] Mannacio V, Di Tommaso L, De Amicis V, Stassano P, Vosa C. Randomized Flow Capacity Comparison of Skeletonized and Pedicled Left Internal Mammary Artery. Ann Thorac Surg 2011;91:24–30. https://doi.org/10.1016/j.athoracsur.2010.06.131.

[24] Aye RW, Swanstrom LL, Kapur S, Buduhan G, Dunst CM, Knight A, et al. A Randomized Multiinstitution Comparison of the Laparoscopic Nissen and Hill Repairs. Ann Thorac Surg 2012;94:951–8. https://doi.org/10.1016/j.athoracsur.2012.04.083.

[25] Licht PB, Pilegaard HK, Ladegaard L. Sympathicotomy for Isolated Facial Blushing: A Randomized Clinical Trial. Ann Thorac Surg 2012;94:401–5. https://doi.org/10.1016/j.athoracsur.2012.03.076.

[26] Raman J, Lehmann S, Zehr K, De Guzman BJ, Aklog L, Garrett HE, et al. Sternal Closure With Rigid Plate Fixation Versus Wire Closure: A Randomized Controlled Multicenter Trial. Ann Thorac Surg 2012;94:1854–61. https://doi.org/10.1016/j.athoracsur.2012.07.085.

[27] Birla R, Twine G, Unsworth-White J. Randomized Trial of Carpentier-Edwards Supraannular Prosthesis Versus Mosaic Aortic Prosthesis: 6 Year Results. Ann Thorac Surg 2013;95:831–7. https://doi.org/10.1016/j.athoracsur.2012.09.031.

[28] Dreifaldt M, Mannion JD, Bodin L, Olsson H, Zagozdzon L, Souza D. The no-touch saphenous vein as the preferred second conduit for coronary artery bypass grafting. Ann Thorac Surg 2013;96:105–11. https://doi.org/10.1016/j.athoracsur.2013.01.102.

[29] Bolotin G, Huber CH, Shani L, Mohr FW, Carrel TP, Borger MA, et al. Novel Emboli Protection System During Cardiac Surgery: A Multi-Center, Randomized, Clinical Trial. Ann Thorac Surg 2014;98:1627–34. https://doi.org/10.1016/j.athoracsur.2014.06.061.

[30] Lee S, Kim HR, Cho S, Huh DM, Lee EB, Ryu KM, et al. Staple Line Coverage After Bullectomy for Primary Spontaneous Pneumothorax: A Randomized Trial. Ann Thorac Surg 2014;98:2005–11. https://doi.org/10.1016/j.athoracsur.2014.06.047.

[31] Min X, Huang Y, Yang Y, Chen Y, Cui J, Wang C, et al. Mechanical Pleurodesis Does Not Reduce Recurrence of Spontaneous Pneumothorax: A Randomized Trial. Ann Thorac Surg 2014;98:1790–6. https://doi.org/10.1016/j.athoracsur.2014.06.034.

[32] Borger MA, Moustafine V, Conradi L, Knosalla C, Richter M, Merk DR, et al. A Randomized Multicenter Trial of Minimally Invasive Rapid Deployment Versus Conventional Full Sternotomy Aortic Valve Replacement. Ann Thorac Surg 2015;99:17–25. https://doi.org/10.1016/j.athoracsur.2014.09.022.

[33] Taggart DP, Ben Gal Y, Lees B, Patel N, Webb C, Rehman SM, et al. A Randomized Trial of External Stenting for Saphenous Vein Grafts in Coronary Artery Bypass Grafting. Ann Thorac Surg 2015;99:2039–45. https://doi.org/10.1016/j.athoracsur.2015.01.060.

[34] Baumbach H, Rustenbach CJ, Ahad S, Nagib R, Albert M, Ratge D, et al. Minimally Invasive Extracorporeal Bypass in Minimally Invasive Heart Valve Operations: A Prospective Randomized Trial. Ann Thorac Surg 2016;102:93–100. https://doi.org/10.1016/j.athoracsur.2016.01.043.

[35] Nguyen BAV, Fiorentino F, Reeves BC, Baig K, Athanasiou T, Anderson JR, et al. Mini Bypass and Proinflammatory Leukocyte Activation: A Randomized Controlled Trial. Ann Thorac Surg 2016;101:1454–63. https://doi.org/10.1016/j.athoracsur.2015.09.029.

[36] Fiorelli A, Messina G, Chiodini P, Costanzo S, Viggiano A, Monda M, et al. Cardiac Autonomic Changes After Thoracic Sympathectomy: A Prospective, Randomized Study. Ann Thorac Surg 2017;103:216–24. https://doi.org/10.1016/j.athoracsur.2016.10.055.

[37] Okami J, Tokunaga T, Kanou T, Kunou H, Ishida D, Fujiwara A, et al. Randomized Study Comparing Equal Height Staples With Graduated Height Staples in Bronchial Closure. Ann Thorac Surg 2017;104:1012–9. https://doi.org/10.1016/j.athoracsur.2017.02.070.

[38] Pettersen Ø, Haram PM, Winnerkvist A, Karevold A, Wahba A, Stenvik M, et al. Pedicled Vein Grafts in Coronary Surgery: Perioperative Data From a Randomized Trial. Ann Thorac Surg 2017;104:1313–7. https://doi.org/10.1016/j.athoracsur.2017.03.076.

[39] Etiwy M, Javadikasgari H, Houghtaling P, Gillinov M. Automated Titanium Fasteners Versus Hand-Tied Knots: A Randomized Controlled Trial. Ann Thorac Surg 2018;106:1160–3. https://doi.org/10.1016/j.athoracsur.2018.05.022.

[40] Long H, Tan Q, Luo Q, Wang Z, Jiang G, Situ D, et al. Thoracoscopic Surgery Versus Thoracotomy for Lung Cancer: Short-Term Outcomes of a Randomized Trial. Ann Thorac Surg 2018;105:386–92. https://doi.org/10.1016/j.athoracsur.2017.08.045.

[41] Salim EF, Ali GA. Impact of Thoracoscopic T2 Sympathectomy on Patients With Primary Palmar and Axillary Hyperhidrosis. Ann Thorac Surg 2018;106:1032–7. https://doi.org/10.1016/j.athoracsur.2018.05.023.

[42] Halfwerk FR, Knol K, Mariani S, Grandjean JG, Mecozzi G. Randomized Trial of Miniaturized Versus Standard Extracorporeal Circulation in Aortic Valve Surgery. Ann Thorac Surg 2019;108:37–44. https://doi.org/10.1016/j.athoracsur.2019.01.019.

[43] Jeong DS, Sung K, Kim WS, Keumhee Cho C, Park PW. Randomized Trial of Concomitant Maze Procedure Using Nitrous Oxide- Versus Argon-Based Cryoablation. Ann Thorac Surg 2019;108:30–6. https://doi.org/10.1016/j.athoracsur.2019.01.005.

[44] Leshnower BG, Rangaraju S, Allen JW, Stringer AY, Gleason TG, Chen EP. Deep Hypothermia With Retrograde Cerebral Perfusion Versus Moderate Hypothermia With Antegrade Cerebral Perfusion for Arch Surgery. Ann Thorac Surg 2019;107:1104–10. https://doi.org/10.1016/j.athoracsur.2018.10.008.

[45] Li B, Xiang J, Zhang Y, Li H, Zhang J, Sun Y, et al. Comparison of Ivor-Lewis vs Sweet Esophagectomy for Esophageal Squamous Cell Carcinoma: A Randomized Clinical Trial. JAMA Surg 2015;150:292. https://doi.org/10.1001/jamasurg.2014.2877.

[46] Damgaard S, Lund JT, Lilleør NB, Perko MJ, Sander K, Dimo B, et al. Comparable three months’ outcome of total arterial revascularization versus conventional coronary surgery: Copenhagen Arterial Revascularization Randomized Patency and Outcome trial. J Thorac Cardiovasc Surg 2008;135:1069–75. https://doi.org/10.1016/j.jtcvs.2007.10.039.

[47] Droghetti A, Schiavini A, Muriana P, Folloni A, Picarone M, Bonadiman C, et al. A prospective randomized trial comparing completion technique of fissures for lobectomy: Stapler versus precision dissection and sealant. J Thorac Cardiovasc Surg 2008;136:383–91. https://doi.org/10.1016/j.jtcvs.2008.04.014.

[48] Falk V, Seeburger J, Czesla M, Borger MA, Willige J, Kuntze T, et al. How does the use of polytetrafluoroethylene neochordae for posterior mitral valve prolapse (loop technique) compare with leaflet resection? A prospective randomized trial. J Thorac Cardiovasc Surg 2008;136:1200–6. https://doi.org/10.1016/j.jtcvs.2008.07.028.

[49] Glineur D, Hanet C, Poncelet A, D’hoore W, Funken J-C, Rubay J, et al. Comparison of saphenous vein graft versus right gastroepiploic artery to revascularize the right coronary artery: A prospective randomized clinical, functional, and angiographic midterm evaluation. J Thorac Cardiovasc Surg 2008;136:482–8. https://doi.org/10.1016/j.jtcvs.2008.01.016.

[50] Guenzinger R, Eichinger WB, Hettich I, Bleiziffer S, Ruzicka D, Bauernschmitt R, et al. A prospective randomized comparison of the Medtronic Advantage Supra and St Jude Medical Regent mechanical heart valves in the aortic position: Is there an additional benefit of supra-annular valve positioning? J Thorac Cardiovasc Surg 2008;136:462–71. https://doi.org/10.1016/j.jtcvs.2007.12.018.

[51] Kamiya H, Akhyari P, Martens A, Karck M, Haverich A, Lichtenberg A. Sternal microcirculation after skeletonized versus pedicled harvesting of the internal thoracic artery: A randomized study. J Thorac Cardiovasc Surg 2008;135:32–7. https://doi.org/10.1016/j.jtcvs.2007.09.004.

[52] Narang S, Satsangi DK, Banerjee A, Geelani MA. Stentless valves versus stented bioprostheses at the aortic position: Midterm results. J Thorac Cardiovasc Surg 2008;136:943–7. https://doi.org/10.1016/j.jtcvs.2008.06.016.

[53] Puskas JD, Halkos ME, Balkhy H, Caskey M, Connolly M, Crouch J, et al. Evaluation of the PAS-Port Proximal Anastomosis System in coronary artery bypass surgery (the EPIC trial). J Thorac Cardiovasc Surg 2009;138:125–32. https://doi.org/10.1016/j.jtcvs.2009.02.017.

[54] Fattouch K, Guccione F, Dioguardi P, Sampognaro R, Corrado E, Caruso M, et al. Off-pump versus on-pump myocardial revascularization in patients with ST-segment elevation myocardial infarction: A randomized trial. J Thorac Cardiovasc Surg 2009;137:650–7. https://doi.org/10.1016/j.jtcvs.2008.11.033.

[55] Formica F, Broccolo F, Martino A, Sciucchetti J, Giordano V, Avalli L, et al. Myocardial revascularization with miniaturized extracorporeal circulation versus off pump: Evaluation of systemic and myocardial inflammatory response in a prospective randomized study. J Thorac Cardiovasc Surg 2009;137:1206–12. https://doi.org/10.1016/j.jtcvs.2008.09.074.

[56] Fattouch K, Guccione F, Sampognaro R, Panzarella G, Corrado E, Navarra E, et al. POINT: Efficacy of adding mitral valve restrictive annuloplasty to coronary artery bypass grafting in patients with moderate ischemic mitral valve regurgitation: A randomized trial. J Thorac Cardiovasc Surg 2009;138:278–85. https://doi.org/10.1016/j.jtcvs.2008.11.010.

[57] Sakwa MP, Emery RW, Shannon FL, Altshuler JM, Mitchell D, Zwada D, et al. Coronary artery bypass grafting with a minimized cardiopulmonary bypass circuit: A prospective, randomized trial. J Thorac Cardiovasc Surg 2009;137:481–5. https://doi.org/10.1016/j.jtcvs.2008.08.057.

[58] Nasso G, Coppola R, Bonifazi R, Piancone F, Bozzetti G, Speziale G. Arterial revascularization in primary coronary artery bypass grafting: Direct comparison of 4 strategies--results of the Stand-in-Y Mammary Study. J Thorac Cardiovasc Surg 2009;137:1093–100. https://doi.org/10.1016/j.jtcvs.2008.10.029.

[59] Belcher E, Dusmet M, Jordan S, Ladas G, Lim E, Goldstraw P. A prospective, randomized trial comparing BioGlue and Vivostat for the control of alveolar air leak. J Thorac Cardiovasc Surg 2010;140:32–8. https://doi.org/10.1016/j.jtcvs.2009.11.064.

[60] Markman PL, Rowland MA, Leong J-Y, Van Der Merwe J, Storey E, Marasco S, et al. Skeletonized internal thoracic artery harvesting reduces chest wall dysesthesia after coronary bypass surgery. J Thorac Cardiovasc Surg 2010;139:674–9. https://doi.org/10.1016/j.jtcvs.2009.03.066.

[61] Ponce González MA, Serdá GJ, Suarez PR, Perez-Peñate G, Gilart JF, Navarro PC. Long-term cardiopulmonary function after thoracic sympathectomy: Comparison between the conventional and simplified techniques. J Thorac Cardiovasc Surg 2010;139:405–10. https://doi.org/10.1016/j.jtcvs.2009.05.011.

[62] Roshanali F, Saidi B, Mandegar MH, Yousefnia MA, Alaeddini F. Echocardiographic approach to the decision-making process for tricuspid valve repair. J Thorac Cardiovasc Surg 2010;139:1483–7. https://doi.org/10.1016/j.jtcvs.2009.08.035.

[63] Salehi Omran A, Karimi A, Ahmadi H, Yazdanifard P, Sheikh Fahtollahi M, Tazik M. Prophylactic ventral cardiac denervation: Does it reduce incidence of atrial fibrillation after coronary artery bypass grafting? J Thorac Cardiovasc Surg 2010;140:1036–9. https://doi.org/10.1016/j.jtcvs.2009.12.024.

[64] Wiklund L, Setina M, Tsang K, Cusimano R, Yau T. A multicenter prospective randomized trial of a second-generation anastomotic device in coronary artery bypass surgery. J Thorac Cardiovasc Surg 2010;139:741–7. https://doi.org/10.1016/j.jtcvs.2009.09.063.

[65] Darling GE, Allen MS, Decker PA, Ballman K, Malthaner RA, Inculet RI, et al. Randomized trial of mediastinal lymph node sampling versus complete lymphadenectomy during pulmonary resection in the patient with N0 or N1 (less than hilar) non–small cell carcinoma: Results of the American College of Surgery Oncology Group Z0030 Trial. J Thorac Cardiovasc Surg 2011;141:662–70. https://doi.org/10.1016/j.jtcvs.2010.11.008.

[66] Al-Rashidi F, Landenhed M, Blomquist S, Höglund P, Karlsson P-A, Pierre L, et al. Comparison of the effectiveness and safety of a new de-airing technique with a standardized carbon dioxide insufflation technique in open left heart surgery: A randomized clinical trial. J Thorac Cardiovasc Surg 2011;141:1128–33. https://doi.org/10.1016/j.jtcvs.2010.07.013.

[67] Bonacchi M, Harmelin G, Peris A, Sani G. A novel strategy to improve systemic oxygenation in venovenous extracorporeal membrane oxygenation: The “χ-configuration.” J Thorac Cardiovasc Surg 2011;142:1197–204. https://doi.org/10.1016/j.jtcvs.2011.01.046.

[68] De Leyn P, Muller M-R, Oosterhuis JWA, Schmid T, Choong CKC, Weder W, et al. Prospective European multicenter randomized trial of PleuraSeal for control of air leaks after elective pulmonary resection. J Thorac Cardiovasc Surg 2011;141:881–7. https://doi.org/10.1016/j.jtcvs.2010.09.019.

[69] Michaux I, Filipovic M, Skarvan K, Bolliger D, Schumann R, Bernet F, et al. A randomized comparison of right ventricular function after on-pump versus off-pump coronary artery bypass graft surgery. J Thorac Cardiovasc Surg 2011;141:361–7. https://doi.org/10.1016/j.jtcvs.2010.02.023.

[70] Speziale G, Nasso G, Esposito G, Conte M, Greco E, Fattouch K, et al. Results of mitral valve repair for Barlow disease (bileaflet prolapse) via right minithoracotomy versus conventional median sternotomy: A randomized trial. J Thorac Cardiovasc Surg 2011;142:77–83. https://doi.org/10.1016/j.jtcvs.2010.08.033.

[71] Benedetto U, Melina G, Angeloni E, Refice S, Roscitano A, Comito C, et al. Prophylactic tricuspid annuloplasty in patients with dilated tricuspid annulus undergoing mitral valve surgery. J Thorac Cardiovasc Surg 2012;143:632–8. https://doi.org/10.1016/j.jtcvs.2011.12.006.

[72] Chaudhuri K, Storey E, Lee GA, Bailey M, Chan J, Rosenfeldt FL, et al. Carbon dioxide insufflation in open-chamber cardiac surgery: A double-blind, randomized clinical trial of neurocognitive effects. J Thorac Cardiovasc Surg 2012;144:646-653.e1. https://doi.org/10.1016/j.jtcvs.2012.04.010.

[73] Lemma MG, Coscioni E, Tritto FP, Centofanti P, Fondacone C, Salica A, et al. On-pump versus off-pump coronary artery bypass surgery in high-risk patients: Operative results of a prospective randomized trial (on-off study). J Thorac Cardiovasc Surg 2012;143:625–31. https://doi.org/10.1016/j.jtcvs.2011.11.011.

[74] Hwang HY, Kim JS, Oh SJ, Kim K-B. A randomized comparison of the Saphenous Vein Versus Right Internal Thoracic Artery as a Y-Composite Graft (SAVE RITA) trial: Early results. J Thorac Cardiovasc Surg 2012;144:1027–35. https://doi.org/10.1016/j.jtcvs.2012.07.054.

[75] Suri RM, Michelena HI, Burkhart HM, Greason KL, Daly RC, Dearani JA, et al. A prospective, randomized comparison of 3 contemporary bioprosthetic aortic valves: Should hemodynamic performance influence device selection? J Thorac Cardiovasc Surg 2012;144:1387–98. https://doi.org/10.1016/j.jtcvs.2012.07.105.

[76] Rogers CA, Pike K, Angelini GD, Reeves BC, Glauber M, Ferrarini M, et al. An open randomized controlled trial of median sternotomy versus anterolateral left thoracotomy on morbidity and health care resource use in patients having off-pump coronary artery bypass surgery: The Sternotomy Versus Thoracotomy (STET) trial. J Thorac Cardiovasc Surg 2013;146:306-316.e9. https://doi.org/10.1016/j.jtcvs.2012.04.020.

[77] Bouchard D, Jensen H, Carrier M, Demers P, Pellerin M, Perrault LP, et al. Effect of systematic downsizing rigid ring annuloplasty in patients with moderate ischemic mitral regurgitation. J Thorac Cardiovasc Surg 2014;147:1471–7. https://doi.org/10.1016/j.jtcvs.2013.05.024.

[78] Svensson LG, Blackstone EH, Apperson-Hansen C, Ruggieri PM, Ainkaran P, Naugle RI, et al. Implications from neurologic assessment of brain protection for total arch replacement from a randomized trial. J Thorac Cardiovasc Surg 2015;150:1140-1147.e11. https://doi.org/10.1016/j.jtcvs.2015.07.054.

[79] Lee R, Vassallo P, Kruse J, Malaisrie SC, Rigolin V, Andrei A-C, et al. A randomized, prospective pilot comparison of 3 atrial appendage elimination techniques: Internal ligation, stapled excision, and surgical excision. J Thorac Cardiovasc Surg 2016;152:1075–80. https://doi.org/10.1016/j.jtcvs.2016.06.009.

[80] Allen KB, Thourani VH, Naka Y, Grubb KJ, Grehan J, Patel N, et al. Randomized, multicenter trial comparing sternotomy closure with rigid plate fixation to wire cerclage. J Thorac Cardiovasc Surg 2017;153:888-896.e1. https://doi.org/10.1016/j.jtcvs.2016.10.093.

[81] Halkos ME, Anderson A, Binongo JNG, Stringer A, Lasanajak Y, Thourani VH, et al. Operative strategies to reduce cerebral embolic events during on- and off-pump coronary artery bypass surgery: A stratified, prospective randomized trial. J Thorac Cardiovasc Surg 2017;154:1278-1285.e1. https://doi.org/10.1016/j.jtcvs.2017.04.089.

[82] Ad N, Holmes SD, Massimiano PS, Rongione AJ, Fornaresio LM, Fitzgerald D. The use of del Nido cardioplegia in adult cardiac surgery: A prospective randomized trial. J Thorac Cardiovasc Surg 2018;155:1011–8. https://doi.org/10.1016/j.jtcvs.2017.09.146.

[83] Marasco SF, Fuller L, Zimmet A, McGiffin D, Seitz M, Ch’ng S, et al. Prospective, randomized, controlled trial of polymer cable ties versus standard wire closure of midline sternotomy. J Thorac Cardiovasc Surg 2018;156:1589-1595.e1. https://doi.org/10.1016/j.jtcvs.2018.04.025.

[84] Nair SK, Sudarshan CD, Thorpe BS, Singh J, Pillay T, Catarino P, et al. Mini-Stern Trial: A randomized trial comparing mini-sternotomy to full median sternotomy for aortic valve replacement. J Thorac Cardiovasc Surg 2018;156:2124-2132.e31. https://doi.org/10.1016/j.jtcvs.2018.05.057.

[85] Wang H, Han J, Wang Z, Yin Z, Liu Z, Jin Y, et al. A prospective randomized trial of the cut-and-sew Maze procedure in patients undergoing surgery for rheumatic mitral valve disease. J Thorac Cardiovasc Surg 2018;155:608–17. https://doi.org/10.1016/j.jtcvs.2017.07.084.

[86] Braathen B, Husebye T, Lunde IG, Tønnessen T. Trifecta has lower gradient and less prosthesis–patient mismatch than Mosaic Ultra in the aortic position: A prospective randomized study. J Thorac Cardiovasc Surg 2019;158:1032–9. https://doi.org/10.1016/j.jtcvs.2018.11.020.

[87] Szwed K, Pawliszak W, Szwed M, Tomaszewska M, Anisimowicz L, Borkowska A. Reducing delirium and cognitive dysfunction after off-pump coronary bypass: A randomized trial. J Thorac Cardiovasc Surg 2019:S0022522319320914. https://doi.org/10.1016/j.jtcvs.2019.09.081.

[88] Jiwnani S, Ranganathan P, Patil V, Agarwal V, Karimundackal G, Pramesh CS. Pain after posterolateral versus nerve-sparing thoracotomy: A randomized trial. J Thorac Cardiovasc Surg 2019;157:380–6. https://doi.org/10.1016/j.jtcvs.2018.07.033.

[89] Suzuki K, Saji H, Aokage K, Watanabe S, Okada M, Mizusawa J, et al. Comparison of pulmonary segmentectomy and lobectomy: Safety results of a randomized trial. J Thorac Cardiovasc Surg 2019;158:895–907. https://doi.org/10.1016/j.jtcvs.2019.03.090.

[90] Slaughter MS, Rogers JG, Milano CA, Russell SD, Conte JV, Feldman D, et al. Advanced Heart Failure Treated with Continuous-Flow Left Ventricular Assist Device. N Engl J Med 2009;361:2241–51. https://doi.org/10.1056/NEJMoa0909938.

[91] Shroyer AL, Grover FL, Hattler B, Collins JF, McDonald GO, Kozora E, et al. On-Pump versus Off-Pump Coronary-Artery Bypass Surgery. N Engl J Med 2009;361:1827–37. https://doi.org/10.1056/NEJMoa0902905.

[92] Jones RH, Velazquez EJ, Michler RE, Sopko G, Oh JK, O’Connor CM, et al. Coronary Bypass Surgery with or without Surgical Ventricular Reconstruction. N Engl J Med 2009;360:1705–17. https://doi.org/10.1056/NEJMoa0900559.

[93] Lamy A, Devereaux PJ, Prabhakaran D, Taggart DP, Hu S, Paolasso E, et al. Off-Pump or On-Pump Coronary-Artery Bypass Grafting at 30 Days. N Engl J Med 2012;366:1489–97. https://doi.org/10.1056/NEJMoa1200388.

[94] Diegeler A, Börgermann J, Kappert U, Breuer M, Böning A, Ursulescu A, et al. Off-pump versus on-pump coronary-artery bypass grafting in elderly patients. N Engl J Med 2013;368:1189–98. https://doi.org/10.1056/NEJMoa1211666.

[95] Acker MA, Parides MK, Perrault LP, Moskowitz AJ, Gelijns AC, Voisine P, et al. Mitral-Valve Repair versus Replacement for Severe Ischemic Mitral Regurgitation. N Engl J Med 2014;370:23–32. https://doi.org/10.1056/NEJMoa1312808.

[96] Smith PK, Puskas JD, Ascheim DD, Voisine P, Gelijns AC, Moskowitz AJ, et al. Surgical Treatment of Moderate Ischemic Mitral Regurgitation. N Engl J Med 2014;371:2178–88. https://doi.org/10.1056/NEJMoa1410490.

[97] Gillinov AM, Gelijns AC, Parides MK, DeRose JJ, Moskowitz AJ, Voisine P, et al. Surgical Ablation of Atrial Fibrillation during Mitral-Valve Surgery. N Engl J Med 2015;372:1399–409. https://doi.org/10.1056/NEJMoa1500528.

[98] Rogers JG, Pagani FD, Tatooles AJ, Bhat G, Slaughter MS, Birks EJ, et al. Intrapericardial Left Ventricular Assist Device for Advanced Heart Failure. N Engl J Med 2017;376:451–60. https://doi.org/10.1056/NEJMoa1602954.

[99] Mehra MR, Naka Y, Uriel N, Goldstein DJ, Cleveland JC, Colombo PC, et al. A Fully Magnetically Levitated Circulatory Pump for Advanced Heart Failure. N Engl J Med 2017;376:440–50. https://doi.org/10.1056/NEJMoa1610426.

[100] Mariette C, Markar SR, Dabakuyo-Yonli TS, Meunier B, Pezet D, Collet D, et al. Hybrid Minimally Invasive Esophagectomy for Esophageal Cancer. N Engl J Med 2019;380:152–62. https://doi.org/10.1056/NEJMoa1805101.

[101] El-Hamamsy I, Eryigit Z, Stevens L-M, Sarang Z, George R, Clark L, et al. Long-term outcomes after autograft versus homograft aortic root replacement in adults with aortic valve disease: a randomised controlled trial. The Lancet 2010;376:524–31. https://doi.org/10.1016/S0140-6736(10)60828-8.

[102] Biere SS, van Berge Henegouwen MI, Maas KW, Bonavina L, Rosman C, Garcia JR, et al. Minimally invasive versus open oesophagectomy for patients with oesophageal cancer: a multicentre, open-label, randomised controlled trial. The Lancet 2012;379:1887–92. https://doi.org/10.1016/S0140-6736(12)60516-9.

[103] Rintoul RC, Ritchie AJ, Edwards JG, Waller DA, Coonar AS, Bennett M, et al. Efficacy and cost of video-assisted thoracoscopic partial pleurectomy versus talc pleurodesis in patients with malignant pleural mesothelioma (MesoVATS): an open-label, randomised, controlled trial. The Lancet 2014;384:1118–27. https://doi.org/10.1016/S0140-6736(14)60418-9.
